# Corticosteroids in septic shock: mechanisms, heterogeneous treatment effects, and trajectory-guided precision pharmacotherapy

**DOI:** 10.3389/fphar.2026.1962609

**Published:** 2026-10-14

**Authors:** Zifei Jia, Xuanyu Zhao, Xiangtian Liu, Xiaoli Li

**Affiliations:** 1 Second Clinical Medical College of Shandong Medical and Pharmaceutical University (Yantai Affiliated Hospital), Yantai, Shandong, China; 2 Department of Critical Care Medicine, Yantai Yuhuangding Hospital, Yantai, Shandong, China

**Keywords:** biomarker enrichment, corticosteroids, fludrocortisone, heterogeneous treatment effects, hydrocortisone, longitudinal trajectories, precision pharmacotherapy, septic shock

## Abstract

Corticosteroid therapy in septic shock has evolved from empirical high-dose anti-inflammatory treatment to low-dose adjunctive pharmacotherapy aimed primarily at restoring vascular responsiveness and accelerating shock reversal. Population-level randomized evidence supports a modest average corticosteroid benefit, most consistently reflected in faster shock reversal and earlier vasopressor liberation, whereas mortality effects remain regimen- and population-dependent. This divergence suggests that average treatment effects alone are insufficient to guide individual decisions. We review how pharmacology, randomized evidence, candidate treatment-effect modifiers, and prespecified longitudinal phenotypes may inform the development of individualized corticosteroid strategies, while defining the evidence required before such strategies can guide clinical care. Glucocorticoid and mineralocorticoid receptor biology provides plausible pathways for both benefit and harm, but no single mechanism, cortisol test, transcriptomic endotype, or clinical trajectory is currently validated for routine treatment selection. Candidate enrichment signals must be distinguished as prognostic, predictive, pharmacodynamic, or safety-related and must be available before corticosteroid exposure when used for treatment selection. Observational studies require explicit alignment of eligibility, time zero, treatment strategies, and follow-up; target trial emulation can reduce avoidable temporal bias but cannot substitute for randomization or remove unmeasured confounding. Trajectory-guided enrichment remains investigational: any candidate trajectory used for treatment selection must be assigned or predicted from information available before exposure, and differential treatment effects require explicit evaluation rather than prognostic association alone. Treatment evaluation should extend beyond mortality to shock reversal, organ-support liberation, kidney outcomes, metabolic toxicity, infection, neuromuscular effects, and longer-term function. Clinical decision support should remain a future, clinician-supervised implementation layer that translates validated evidence into time-stamped actions rather than an autonomous prescribing system. Progress toward precision corticosteroid therapy now requires prespecified enrichment hypotheses, direct regimen comparisons, harmonized outcomes, prospective validation, and trials demonstrating clinical utility.

## 1 Introduction

Sepsis is defined as life-threatening organ dysfunction caused by a dysregulated host response to infection, while septic shock denotes a severe circulatory and metabolic phenotype associated with a high risk of death (Singer et al., 2016). Despite advances in antimicrobial therapy, source control, hemodynamic resuscitation, and organ support, a substantial proportion of patients remain dependent on vasopressors and develop progressive multiorgan dysfunction. Contemporary guidelines continue to place corticosteroids within the management of adult septic shock rather than unselected sepsis, but the recommendation is conditional and does not provide a validated individualized initiation rule (Chaudhuri et al., 2024a; Prescott et al., 2026).

The scientific interpretation of corticosteroids has changed repeatedly. Early high-dose, short-course regimens sought to suppress systemic inflammation rapidly but failed to improve survival and raised concern about treatment-related harm. Later low-dose hydrocortisone strategies shifted the therapeutic objective toward restoration of vascular responsiveness and shock reversal. CORTICUS and ADRENAL showed faster hemodynamic recovery without a mortality benefit, whereas APROCCHSS demonstrated lower mortality with a hydrocortisone plus fludrocortisone regimen in a population with more severe shock (Sprung et al., 2008; Venkatesh et al., 2018; Annane et al., 2018). These trials define a clinically useful average treatment effect, but they do not establish that every eligible patient derives the same benefit or faces the same risk.

This unresolved heterogeneity is central to pharmacotherapy. Corticosteroids can modulate inflammatory transcription, endogenous cortisol signaling, vascular responsiveness, glucose and electrolyte homeostasis, and immune function. The same exposure may therefore accelerate vasopressor liberation in one patient while producing limited benefit or clinically important toxicity in another. Differences in shock burden, infection control, comorbidity, host-response state, receptor responsiveness, drug choice, administration mode, timing, and duration may all contribute. A precision approach must consequently move beyond the question of whether corticosteroids work on average and ask which treatment version is most likely to produce a favorable net effect in a defined patient at a defined decision time.

In this review, precision pharmacotherapy is defined operationally as the use of patient information available at a prespecified treatment-decision time to estimate the expected net benefit–harm contrast among clinically implementable corticosteroid strategies. Four distinct functions should be separated. Risk prediction estimates the probability of an outcome irrespective of whether relative treatment effects differ; treatment-effect prediction estimates how outcomes may differ under alternative treatment strategies; treatment selection applies sufficiently validated treatment-effect evidence to choose among clinically relevant strategies; and response monitoring evaluates physiological benefit, treatment failure, or toxicity after exposure. These functions are not interchangeable. In particular, a high-risk patient is not necessarily more responsive to corticosteroids, and a post-treatment improvement in vasopressor dose, mean arterial pressure, or lactate cannot retrospectively serve as a pretreatment predictor of benefit.

Clinical phenotypes and transcriptomic endotypes have provided candidate explanations for treatment-effect heterogeneity. Data-driven sepsis phenotypes differ in organ dysfunction and prognosis, and secondary analyses of randomized trials have suggested that host-response signatures may modify corticosteroid effects (Seymour et al., 2019; Antcliffe et al., 2019). However, prognostic risk is not equivalent to predictive enrichment, classifiers developed in different cohorts are not interchangeable, and several reported interactions remain exploratory or have required correction. Accordingly, clinical phenotypes and transcriptomic endotypes currently provide hypothesis-generating enrichment signals rather than validated treatment-selection rules, and no molecular classifier is clinically qualified as a companion diagnostic for corticosteroid use.

Longitudinal electronic health record data add a temporal dimension that is absent from a single baseline score. Vasopressor dose, mean arterial pressure, lactate, organ dysfunction, fluid balance, and kidney function evolve together and may better represent the clinical state in which a corticosteroid decision is made. Yet trajectories are also shaped by treatment and measurement processes. Post-exposure improvement cannot be used retrospectively to define pretreatment benefit, and observational comparisons are vulnerable to immortal time, confounding by indication, and time-varying confounding. Recent work combining predicted organ-dysfunction trajectories with target trial emulation illustrates a pathway for hypothesis generation, but not a substitute for randomized evidence (Rajendran et al., 2025). Critically, an observed post-treatment trajectory and a trajectory class predicted from pretreatment information have different inferential roles and should not be used interchangeably for treatment selection. Accordingly, current trajectory-based and observational causal studies should be regarded as hypothesis-generating evidence rather than validated treatment-selection evidence.

This review therefore organizes corticosteroid precision pharmacotherapy according to evidentiary readiness for treatment selection rather than methodological complexity. Tier one comprises established clinical evidence from randomized trials and contemporary guidelines that defines population-level eligibility and tested regimen-level effects. Tier 2 comprises evidence that is biologically or clinically supported but remains uncertain for individualized prescribing, including cortisol physiology, receptor biology, treatment-version differences, dose–exposure relationships, timing, duration, and withdrawal strategies. Tier 3 comprises hypothesis-generating evidence from clinical phenotypes, transcriptomic endotypes, treatment-effect interaction analyses, longitudinal trajectories, and observational causal studies. Tier 4 comprises proposed research and implementation frameworks, including prospectively validated trajectory-guided enrichment, individualized treatment-effect modeling, target-trial design strategies, and clinician-supervised decision support.

These tiers indicate current clinical evidentiary readiness, not a hierarchy of methodological sophistication. Increasing analytical complexity does not confer stronger causal validity or greater readiness for bedside use. Randomized evidence remains the foundation for current corticosteroid treatment, whereas supported but uncertain evidence identifies unresolved regimen and pharmacological questions, hypothesis-generating evidence defines candidates for prospective testing, and proposed research frameworks describe how future individualized strategies might be validated and implemented. Figure 1 provides historical context for the changing therapeutic questions and study designs that shaped contemporary corticosteroid use, whereas Table 1 summarizes this four-tier evidence-readiness framework. The aim of the review is therefore not to present a bedside-ready prescribing algorithm, but to define what is currently established, what remains uncertain, which precision signals warrant prospective testing, and what evidence would be required before individualized corticosteroid strategies could guide clinical care.

**FIGURE 1:**
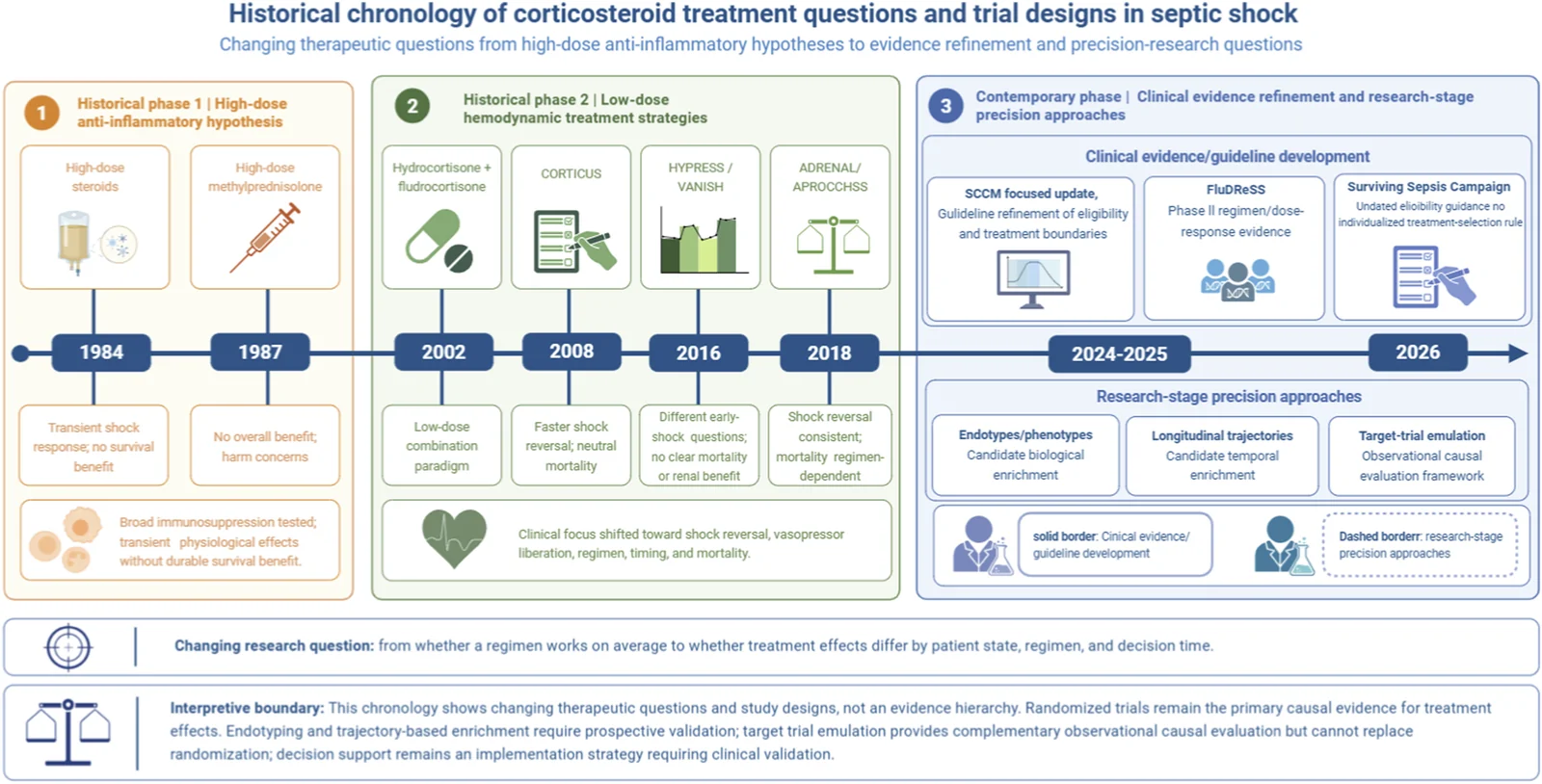
Historical chronology of corticosteroid treatment questions and trial designs in septic shock. The chronology illustrates changing therapeutic questions and study designs rather than a hierarchy of evidence. Contemporary research incorporates enrichment, causal evaluation, and implementation approaches, but these remain complementary frameworks requiring validation. The 2024–2025 period includes the SCCM focused update, FluDReSS, endotype and trajectory studies, and target trial emulation; the 2026 period reflects updated Surviving Sepsis Campaign eligibility guidance. Precision-oriented approaches remain a research program rather than a validated prescribing rule. The 2016 node separates HYPRESS, which tested prophylaxis before shock, from VANISH, which enrolled patients early after septic-shock onset.

**TABLE 1:** Four-tier evidence-readiness framework for precision corticosteroid pharmacotherapy in septic shock.

Evidence level	Evidence domain and representative evidence	What the evidence currently supports	What it does not establish	Implication for precision treatment
Tier 1. Established clinical evidence	Major randomized trials and contemporary guideline recommendations, including CORTICUS, ADRENAL, APROCCHSS, VANISH, COIITSS, HYPRESS, and current critical-care guidance	Contemporary low-dose corticosteroid regimens can accelerate shock reversal and vasopressor liberation in adults with established septic shock. Mortality effects are regimen- and population-dependent, with benefit demonstrated for some treatment versions but not uniformly across trials. High-dose, short-course anti-inflammatory regimens and prophylactic hydrocortisone before shock are not supported	Does not identify an individual corticosteroid responder, define a universal vasopressor threshold for initiation, establish an optimal duration or withdrawal strategy, or demonstrate that all corticosteroid regimens are interchangeable	Current clinical evidence is sufficient to define population-level eligibility and tested regimen-level effects, but not individualized treatment selection
Tier 2. Supported but uncertain evidence	Cortisol physiology and CIRCI; GR/MR biology; vascular and pharmacodynamic studies; hydrocortisone monotherapy versus hydrocortisone plus fludrocortisone pharmacology; dose–exposure, administration-mode, duration, and withdrawal evidence	Provides biological and pharmacological plausibility for heterogeneity in corticosteroid response and supports several clinically relevant regimen-level hypotheses. Direct and pooled evidence can inform the likely effects and harms of different treatment versions	Does not establish a validated cortisol-, receptor-, dose-, timing-, or toxicity-based rule for choosing corticosteroids in an individual patient. The incremental contribution of fludrocortisone and the optimal dose, timing, administration mode, duration, and withdrawal strategy remain uncertain	Clinically relevant and biologically supported, but insufficient for individualized prescribing without further direct comparative evidence
Tier 3. Hypothesis-generating evidence	Clinical severity and phenotype studies; transcriptomic endotypes including SRS1/SRS2, pediatric Endotype A/B, IA-P/IN-P and CTS; treatment-by-marker analyses; longitudinal SOFA, lactate and hemodynamic trajectories; observational treatment-effect studies and target-trial emulations	Identifies candidate biological or longitudinal states that may modify treatment benefit or harm and provides testable hypotheses for predictive enrichment. Formal treatment-by-marker or treatment-by-trajectory interactions can generate evidence of possible heterogeneous treatment effects	Prognostic risk alone does not establish predictive enrichment. Secondary, *post hoc*, observational, or corrected subgroup signals do not establish a validated treatment-selection biomarker. Observational TTE cannot eliminate unmeasured confounding or prove that earlier treatment, a particular trajectory, or a specific regimen is superior	Suitable for prospective hypothesis testing and enrichment research; not ready for routine corticosteroid selection
Tier 4. Proposed research and implementation framework	Prespecified trajectory-guided enrichment; prospective treatment-effect validation; target-trial design principles; multimodal prediction; clinician-supervised CDSS; reinforcement learning and AI implementation frameworks	Provides a structured pathway for testing individualized corticosteroid strategies, separating pretreatment selection from post-treatment response, evaluating multidimensional benefit–harm, and ultimately translating validated evidence into clinician-supervised decisions	Does not constitute evidence that individualized corticosteroid treatment improves outcomes. No corticosteroid-specific trajectory rule, AI model, reinforcement-learning policy, or CDSS has established prospective clinical utility for routine treatment selection	Research and future implementation only; not a validated clinical decision-making framework

The four tiers reflect evidentiary readiness for corticosteroid treatment selection rather than methodological sophistication. A method may contribute to more than one tier depending on its role. For example, observational estimates obtained from target-trial emulation remain hypothesis-generating, whereas target-trial principles can also provide a framework for designing future causal evaluations. Increasing analytical complexity does not imply greater clinical validity or readiness.

## 2 Approach to evidence identification and synthesis

This article is a state-of-the-art narrative review informed by structured evidence identification rather than a systematic review, scoping review, or meta-analysis (Grant and Booth, 2009). The review was designed to synthesize representative evidence and to develop a clinically testable pharmacological framework. Evidence identification was organized around six domains: receptor and drug pharmacology; randomized trials, guidelines, and safety syntheses; heterogeneous treatment effects and biomarker enrichment; longitudinal trajectories and causal treatment evaluation; organ-specific benefit-harm outcomes; and clinical decision-support validation and implementation. Searches were conducted in PubMed/MEDLINE, Embase, and the Cochrane Central Register of Controlled Trials from inception to June 2026 using combinations of “septic shock,” “corticosteroids,” “hydrocortisone,” “fludrocortisone,” “transcriptomics,” “biomarker,” “trajectory,” and “target trial emulation”; targeted guideline and regulatory searches supplemented these databases.

Priority was given to pivotal randomized trials, contemporary clinical guidelines, systematic reviews, human physiological and translational studies, externally evaluated phenotype or endotype studies, and methodological papers directly relevant to treatment timing, causal inference, outcome construction, transportability, or clinical implementation. Mechanistic evidence was classified as clinical, translational human, or preclinical/molecular. Biomarkers and clinical variables were interpreted according to their intended role as prognostic, predictive, pharmacodynamic-response, or safety indicators. Where findings were secondary, exploratory, *post hoc*, or observational, that status was retained in the synthesis.

Evidence was integrated narratively according to its function in the proposed framework rather than pooled quantitatively. Particular attention was paid to treatment-version heterogeneity, the temporal availability of candidate enrichment variables, separation of pretreatment and post-treatment information, and the distinction between risk prediction and causal treatment-effect estimation. This approach does not provide exhaustive study capture or formal risk-of-bias scoring. It is intended to clarify current evidence boundaries, identify contradictions that require prospective testing, and define a research pathway for trajectory-guided precision pharmacotherapy.

## 3 Pharmacological foundations of corticosteroid therapy

The pharmacological rationale for corticosteroids in septic shock extends beyond nonspecific suppression of inflammation. Hydrocortisone can influence inflammatory transcription, vascular responsiveness, endogenous cortisol signaling, and electrolyte and metabolic homeostasis. These effects are not uniform across tissues or patients and may depend on receptor biology, illness phase, drug exposure, and concomitant organ dysfunction. Accordingly, mechanistic evidence should be interpreted across three levels: direct clinical pharmacodynamic observations in septic shock, translational evidence from critically ill patients or human experimental models, and preclinical molecular evidence. This distinction is essential because several commonly cited mechanisms are biologically plausible but have not been directly established in human septic shock.

### 3.1 Glucocorticoid and mineralocorticoid receptor biology

Hydrocortisone enters target cells and binds the cytosolic glucocorticoid receptor (GR), which is maintained in an inactive multiprotein complex before ligand-dependent conformational change and nuclear translocation. Activated GR regulates transcription through several mechanisms, including direct DNA binding, interaction with other transcriptional regulators, and induction of anti-inflammatory or transcription-repressive proteins. The traditional separation of GR action into transactivation and transrepression remains useful descriptively, but it should not be treated as a complete mechanistic model. Experimental studies demonstrate that GR-mediated repression is gene- and context-dependent and can occur at different stages of the transcription cycle (Scheinman et al., 1995; King et al., 2013; Weikum et al., 2017; Sacta et al., 2018).

Mineralocorticoid receptor (MR) biology is also relevant because receptor selectivity is determined not only by ligand affinity but also by tissue-specific prereceptor metabolism. In classic mineralocorticoid target tissues, 11β-hydroxysteroid dehydrogenase type 2 converts cortisol to cortisone and thereby limits cortisol access to MR. This protective mechanism is not uniform across vascular, immune, and renal tissues, so the net MR effect of hydrocortisone cannot be inferred from *in vitro* affinity alone (Hirasawa et al., 1997; Ferrari and Krozowski, 2000; Dailymed, 2026a). Moreover, receptor abundance is not equivalent to receptor function. Small human and translational studies in sepsis have reported changes in GRα/GRβ expression and serum-induced glucocorticoid resistance, whereas other cohorts have linked higher GR expression to worse outcomes. Together, these findings argue against a universal model of GR downregulation or a single systemic corticosteroid-resistance phenotype (Guerrero et al., 2013; Vardas et al., 2017; Teblick et al., 2022).

### 3.2 Genomic regulation of inflammation and immunity

The anti-inflammatory actions of corticosteroids include suppression of cytokine and chemokine transcription, modulation of leukocyte trafficking, and attenuation of endothelial and immune-cell activation. GR can inhibit nuclear factor kappa B (NF-κB)-dependent transcription and interact with activator protein 1 (AP-1)-related regulatory programs, but these effects are not mediated by a single universal tethering mechanism. GR can directly bind selected AP-1-related DNA motifs, and the consequences of receptor activation vary according to chromatin context, target gene, and cellular state (Scheinman et al., 1995; King et al., 2013; Weikum et al., 2017; Sacta et al., 2018). Some inflammatory genes are repressed directly, whereas others require newly synthesized regulatory proteins, illustrating that glucocorticoid action combines rapid transcriptional interference with transactivation-dependent anti-inflammatory programs (King et al., 2013).

This mechanistic complexity is particularly important in sepsis, where inflammatory activation and immune dysfunction may coexist. A reduction in inflammatory mediator expression may support vascular stabilization and limit tissue injury, but the same exposure can also impair pathogen clearance or deepen an immunosuppressive state in susceptible patients. Therefore, genomic anti-inflammatory activity should not be equated with predictable net clinical benefit. The relevant pharmacological question is not whether hydrocortisone suppresses inflammatory transcription, but whether the magnitude and timing of that suppression are appropriate for a given host-response state.

### 3.3 Rapid and non-genomic cardiovascular effects

The most reproducible clinical effect of low-dose corticosteroids in septic shock is accelerated hemodynamic stabilization, which provides an important bridge between molecular pharmacology and bedside outcomes. Small human physiological studies showed that intravenous hydrocortisone enhanced the pressor response to norepinephrine or phenylephrine in patients with septic shock (Annane et al., 1998; Bellissant and Annane, 2000). In a randomized crossover study, hydrocortisone increased mean arterial pressure and systemic vascular resistance while reducing heart rate, cardiac index, and norepinephrine requirements. Changes in circulating nitrate and nitrite concentrations were associated with reduced vasopressor use, supporting–without proving–involvement of the nitric oxide pathway (Keh et al., 2003).

The rapid time course of these physiological responses is compatible with a non-genomic contribution, but human septic-shock studies have not established a specific membrane GR, kinase, or ion-channel pathway. Restoration of catecholamine responsiveness may involve adrenergic signaling, post-receptor contractile mechanisms, and regulation of inducible nitric oxide synthase rather than a uniform increase in α1-or β1-adrenoceptor expression. Healthy-volunteer studies conducted under experimentally induced hypoaldosteronism have produced different vascular responses to hydrocortisone and fludrocortisone, further indicating that corticosteroid pharmacodynamics depend on the underlying physiological state (Laviolle et al., 2013; Laviolle et al., 2010). Direct restoration of endothelial barrier integrity is biologically plausible and supported mainly by experimental evidence; it has not been established as a clinical mechanism in human septic shock. These pathways remain translational or preclinical hypotheses; none has been shown to mediate hydrocortisone-induced shock reversal in patients.

At the molecular level, preclinical cardiovascular models have described rapid GR-associated phosphatidylinositol 3-kinase (PI3K)-Akt-endothelial nitric oxide synthase (eNOS) signaling after pharmacological corticosteroid exposure (Hafezi-Moghadam et al., 2002). Glucocorticoid regulation of endothelial caveolin-1 has also been demonstrated in cultured vascular cells, although this effect develops on a transcriptional timescale rather than constituting a proven rapid vasopressor mechanism (Igarashi et al., 2013). These pathways deepen the molecular plausibility of vascular corticosteroid effects, but neither has been established as the mechanism responsible for hydrocortisone-induced shock reversal in human septic shock.

### 3.4 Cortisol availability, metabolism, and critical illness-related corticosteroid insufficiency

Critical illness-related corticosteroid insufficiency (CIRCI) cannot be reduced to inadequate adrenal secretion or a single low serum cortisol concentration. Three related but non-equivalent concepts should be distinguished: CIRCI as a clinical diagnostic construct, critical-illness alterations in systemic cortisol availability or tissue glucocorticoid responsiveness as pathophysiological states, and clinical responsiveness to exogenous corticosteroid therapy as a treatment-effect question (Annane et al., 2017). Critical illness alters cortisol-binding globulin and albumin concentrations, so total serum cortisol may underestimate free cortisol availability in patients with low binding-protein levels (Hamrahian et al., 2004). At the same time, elevated circulating cortisol during critical illness may reflect reduced hepatic and renal inactivation and impaired clearance rather than a proportionate increase in secretion. In a human metabolic study, circulating cortisol concentrations were approximately 3.5-fold higher in critically ill patients than in controls, while cortisol production was only moderately increased and clearance was substantially reduced (Boonen et al., 2013).

Cortisol-cortisone interconversion and 11β-hydroxysteroid dehydrogenase activity may also change during critical illness, but the apparent direction of these changes depends on the tissue studied, the phase of illness, and whether investigators use metabolite ratios or direct clearance measurements (Boonen et al., 2013; Venkatesh et al., 2007). Systemic hormone availability must therefore be distinguished from tissue responsiveness. Small sepsis studies and translational models support the biological plausibility of altered GR isoform expression and tissue glucocorticoid resistance, but no peripheral-blood receptor measurement has been validated as a bedside treatment-selection test (Guerrero et al., 2013; Vardas et al., 2017; Teblick et al., 2022). Consistent with this complexity, the 2017 SCCM/ESICM CIRCI guidance did not endorse a single diagnostic test that reliably identifies corticosteroid-responsive patients (Annane et al., 2017). The 2024 SCCM focused update revisited corticosteroid treatment recommendations for sepsis, acute respiratory distress syndrome, and community-acquired pneumonia, but did not establish a new cortisol- or corticotropin-based diagnostic threshold for identifying individual corticosteroid responders in septic shock (Chaudhuri et al., 2024a). No random cortisol or corticotropin-stimulation threshold has been prospectively validated to identify individual hydrocortisone responders in septic shock.

Beyond systemic cortisol availability, prereceptor metabolism may create tissue-specific glucocorticoid exposure. 11β-HSD1 predominantly regenerates active cortisol from cortisone and is induced during human monocyte-to-macrophage differentiation, providing a mechanism for amplification of local glucocorticoid signaling in inflammatory tissues (Thieringer et al., 2001). This regulation is not uniform across disease states: impaired alveolar-macrophage 11β-HSD1 reductase activity has been reported in sepsis-related ARDS (Mahida et al., 2023). Altered local glucocorticoid activation is therefore a plausible contributor to heterogeneous corticosteroid sensitivity rather than a universal sepsis phenotype. Neither 11β-HSD1 activity nor the GRα/GRβ ratio has been validated as a real-time bedside biomarker for corticosteroid selection or precision dosing.

### 3.5 Hydrocortisone and fludrocortisone: complementary pharmacology or unresolved incremental benefit?

Hydrocortisone provides glucocorticoid activity with clinically relevant salt-retaining properties, whereas fludrocortisone has potent and prolonged mineralocorticoid-like effects, including sodium retention and increased urinary potassium excretion. Official pharmacology information reports a biological half-life of approximately 18–36 h for fludrocortisone, but these properties do not by themselves establish a survival benefit in septic shock (Dailymed, 2026a; Dailymed, 2026b). The potential rationale for combination therapy is that additional mineralocorticoid activity could complement GR-mediated anti-inflammatory and vascular effects. However, the clinical contribution of this component remains unresolved.

MR is expressed in vascular endothelial and smooth-muscle cells (Ibarrola and Jaffe, 2024). In murine endotoxemia and complementary human vascular-cell experiments, aldosterone restored vascular MR and α1-adrenoceptor expression and improved vascular reactivity, providing a translational rationale for mineralocorticoid signaling in vasoplegia (Fadel et al., 2017). These findings remain preclinical or translational: vascular-cell-specific MR signaling has not been established as the mechanism of fludrocortisone benefit in human septic shock.

COIITSS provides direct component-level randomized evidence. In this multicenter 2 × 2 factorial trial of 509 adults with septic shock receiving hydrocortisone, adding fludrocortisone did not significantly reduce in-hospital mortality (42.9% vs. 45.8%; RR 0.94, 95% CI 0.77-1.14; P = 0.50) or improve vasopressor- or ventilation-free days (COIITSS Study Investigators, 2010). Because the fludrocortisone comparison was a secondary objective and was not included in the sample-size calculation, a modest incremental effect could not be excluded. APROCCHSS later showed lower mortality for the complete regimen of hydrocortisone plus fludrocortisone versus placebo, whereas cross-trial comparison with ADRENAL cannot isolate the fludrocortisone component (Venkatesh et al., 2018; Annane et al., 2018). FluDReSS showed variable enteral exposure without faster shock resolution, and observational data suggest possible benefit but remain confounded (Walsham et al., 2024; Bosch et al., 2023). Thus, the incremental mineralocorticoid contribution has been tested directly but remains clinically inconclusive.

### 3.6 Exposure, duration, and treatment-related harm

The net effect of corticosteroid therapy reflects both pharmacological intensity and host susceptibility. Hydrocortisone can cause hyperglycemia, sodium and fluid retention, potassium loss, hypothalamic-pituitary-adrenal axis suppression, and immunosuppression with increased infection risk; several of these effects become more likely with higher or more prolonged exposure (Dailymed, 2026a). Trial data confirm that metabolic toxicity is clinically relevant even with contemporary regimens. In HYPRESS, hydrocortisone did not prevent progression to septic shock in patients with severe sepsis without shock and increased hyperglycemia (90.9% vs. 81.5%) (Keh et al., 2016). In APROCCHSS, hyperglycemia was more frequent with the hydrocortisone plus fludrocortisone regimen (Annane et al., 2018). At the population level, a 2024 systematic review and dose-response meta-analysis of 45 randomized trials involving 9,563 patients found that corticosteroids probably reduced short-term mortality (RR 0.93, 95% CI 0.88–0.99) and increased shock reversal at 7 days (RR 1.24, 95% CI 1.11–1.38) (Pitre et al., 2024). Its dose-response model suggested the largest modeled mortality reduction at approximately 260 mg/day of hydrocortisone equivalent (RR 0.90, 95% CI 0.83–0.98) (Pitre et al., 2024). This estimate should be interpreted as a trial-level modeled dose-response relationship rather than a validated individual optimal dose, because the contributing trials differed in corticosteroid molecule, regimen, treatment duration, illness severity, and background care (Pitre et al., 2024).

CORTICUS further illustrates why hemodynamic response and net clinical benefit must be separated. Hydrocortisone accelerated shock reversal without improving survival. In the per-protocol safety population, hyperglycemia occurred in 85% versus 72% and hypernatremia in 29% versus 18% of participants receiving hydrocortisone and placebo, respectively. Superinfection and recurrent-shock events were numerically more frequent, although several infectious estimates were imprecise (Sprung et al., 2008). Administration mode may also modify exposure patterns: a small randomized study found similar mean glucose concentrations with continuous and intermittent hydrocortisone, but more hyperglycemic excursions and insulin adjustments with bolus dosing (Loisa et al., 2007). This finding does not establish a clinical-outcome advantage for continuous infusion.

Neuromuscular weakness remains a biologically plausible corticosteroid-related harm, but its causal magnitude with short-course, low-dose septic-shock regimens is uncertain because available studies use heterogeneous definitions and are frequently underpowered for this outcome (Yang et al., 2018). More broadly, although aggregate trial-level dose-response relationships have been modeled, no dose-exposure relationship has been prospectively validated to individualize corticosteroid dosing or predict toxicity in patients with septic shock (Pitre et al., 2024). The benefit–harm relationship should therefore be represented conceptually rather than with an assumed threshold: increasing or prolonged exposure may strengthen anti-inflammatory and vascular effects while simultaneously increasing metabolic, electrolyte, infectious, and neuromuscular toxicity. These competing pathways provide a mechanistic basis for evaluating treatment response and harm dynamically rather than assuming that a fixed regimen produces uniform benefit. Figure 2 summarizes the evidence-graded balance between potential beneficial pathways and treatment-related harm, while Table 2 specifies the principal mechanism–effect–evidence boundaries.

**FIGURE 2:**
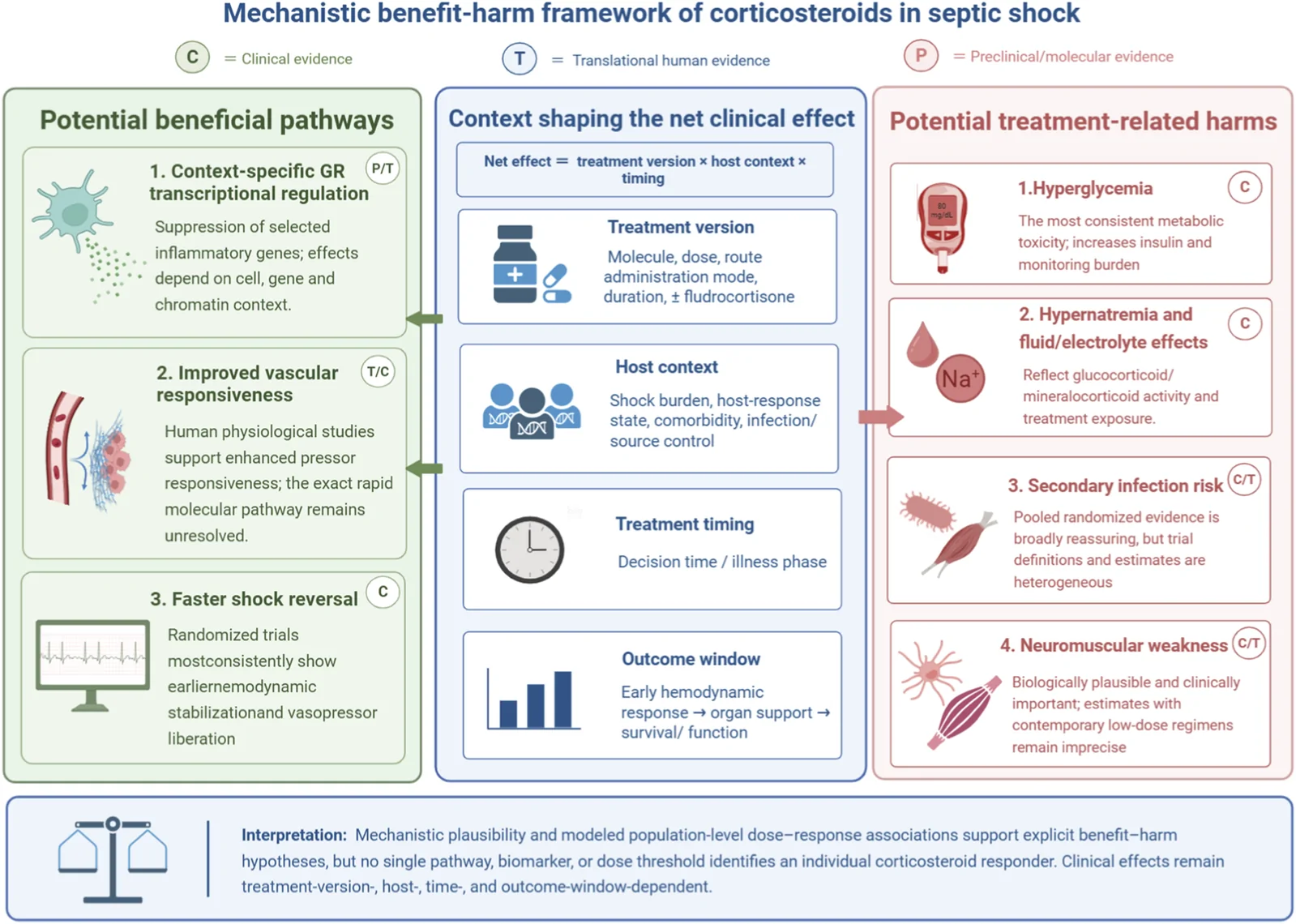
Mechanistic benefit–harm balance of corticosteroids in septic shock. Potential benefit arises from context-dependent glucocorticoid signaling, inflammatory modulation, and restoration of vascular responsiveness, while harm may involve metabolic, electrolyte, immune, and neuromuscular effects. Evidence markers distinguish direct clinical support from translational-human and preclinical/molecular evidence; mechanistic plausibility does not establish an individual treatment effect. Net clinical effects are shaped by treatment version, host context, timing, and outcome window.

**TABLE 2:** Mechanism–effect–evidence matrix for corticosteroid pharmacology in septic shock.

Mechanism or pathway	Proposed pharmacological effect	Evidence level and representative sources	Potential clinical correlate	Main uncertainty or interpretive boundary
GR activation and nuclear signaling	Direct DNA binding, co-regulator recruitment, and induction or repression of context-specific transcriptional programs	Preclinical/molecular; human cell and chromatin studies (Scheinman et al., 1995; King et al., 2013; Weikum et al., 2017; Sacta et al., 2018)	Reduced inflammatory mediator expression and altered immune-cell activation	Not a single transrepression pathway; gene-, cell-, and chromatin-context dependent
NF-κB and AP-1 crosstalk	Suppression of selected inflammatory genes through direct and indirect transcriptional mechanisms	Preclinical/molecular (Scheinman et al., 1995; King et al., 2013; Weikum et al., 2017; Sacta et al., 2018)	Biological basis for anti-inflammatory activity	Does not establish net benefit in septic shock or identify individual responders
GR expression, isoforms, and tissue responsiveness	Altered receptor abundance, isoform balance, or downstream signaling may modify corticosteroid sensitivity	Translational human plus *in vitro*/animal evidence (Guerrero et al., 2013; Vardas et al., 2017; Teblick et al., 2022)	Potential explanation for heterogeneous pharmacological response	Expression is not equivalent to function; no validated bedside GR-resistance phenotype
MR signaling and 11β-HSD1/2	11β-HSD1 regenerates active cortisol from cortisone, whereas 11β-HSD2 limits cortisol access to MR in selected tissues; vascular MR signaling may modify vasomotor responsiveness	Translational/molecular physiology (Hirasawa et al., 1997; Thieringer et al., 2001; Mahida et al., 2023; Ibarrola and Jaffe, 2024; Fadel et al., 2017)	May contribute to tissue-specific glucocorticoid sensitivity, vascular responsiveness, and sodium/fluid effects	Regulation is tissue- and disease-state dependent; neither 11β-HSD1 activity nor vascular MR signaling is a validated bedside treatment-selection biomarker
Catecholamine responsiveness	Improved pressor responsiveness through adrenergic and post-receptor vascular mechanisms	Small human septic-shock pharmacodynamic studies (Annane et al., 1998; Bellissant and Annane, 2000)	Higher MAP and reduced vasopressor requirement	Small samples and surrogate endpoints; no uniform alpha1/beta1 receptor-expression mechanism established
Nitric oxide and vascular tone	Modulation of excessive NO-related vasodilation and vascular smooth-muscle responsiveness	Human randomized crossover evidence with indirect pathway markers (Keh et al., 2003)	Increased systemic vascular resistance and lower norepinephrine requirement	Circulating nitrate/nitrite changes do not prove direct endothelial iNOS inhibition
Rapid or non-genomic effects	Rapid membrane-proximal signaling may alter vascular function; preclinical GR-associated PI3K-Akt-eNOS signaling provides one mechanistic model, while endothelial caveolin-1 regulation illustrates additional receptor crosstalk on a slower transcriptional timescale	Human septic-shock time-course observations plus preclinical/cellular evidence (Annane et al., 1998; Bellissant and Annane, 2000; Keh et al., 2003; Hafezi-Moghadam et al., 2002; Igarashi et al., 2013)	Potential contribution to early hemodynamic response after corticosteroid exposure	No specific membrane-GR, PI3K-Akt-eNOS, caveolin, kinase, or ion-channel pathway has been established as the mechanism of shock reversal in human septic shock
Endothelial barrier and microcirculation	Potential stabilization of endothelial permeability and microvascular function	Predominantly preclinical/translational	Possible reduction in vasoplegia or capillary leak	Direct endothelial barrier restoration has not been demonstrated in human septic shock
Binding proteins and free cortisol	Changes in albumin and cortisol-binding globulin alter the relationship between total and free cortisol	Human critical-illness physiology (Hamrahian et al., 2004)	Explains why low total cortisol may not indicate low biologically available cortisol	Free cortisol is not a validated predictor of treatment benefit
Cortisol metabolism and clearance	Reduced hepatic and renal inactivation can sustain hypercortisolemia despite only modestly increased production	Human mechanistic studies (Boonen et al., 2013; Venkatesh et al., 2007)	Reframes CIRCI beyond adrenal secretion failure	Direction and magnitude depend on method, tissue, and illness phase
CIRCI and tissue glucocorticoid resistance	Mismatch between systemic cortisol availability and tissue corticosteroid action	Guideline plus human translational evidence (Guerrero et al., 2013; Vardas et al., 2017; Teblick et al., 2022; Annane et al., 2017; Hamrahian et al., 2004; Boonen et al., 2013; Venkatesh et al., 2007)	Potential contributor to persistent vasopressor-dependent shock	No cortisol, corticotropin, or receptor threshold reliably identifies responders
Hydrocortisone pharmacology	GR-mediated anti-inflammatory and vascular effects with clinically relevant salt-retaining activity	Official pharmacology plus septic-shock RCTs (Sprung et al., 2008; Venkatesh et al., 2018; Dailymed, 2026a)	Shock reversal and vasopressor liberation	Mortality benefit is not uniform; metabolic and electrolyte toxicity remains relevant
Fludrocortisone pharmacology	Additional potent mineralocorticoid-like activity with sodium retention and potassium excretion; vascular MR signaling provides a plausible complementary vasomotor mechanism	Official pharmacology, translational vascular evidence, regimen-level RCT, phase II PK/PD, and observational evidence (Annane et al., 2018; Dailymed, 2026b; Ibarrola and Jaffe, 2024; Fadel et al., 2017; Walsham et al., 2024; Bosch et al., 2023)	Possible complement to hydrocortisone in severe vasoplegic shock	Incremental component effect remains unresolved; enteral exposure is variable, and vascular MR signaling has not been established as the mechanism of clinical benefit
Metabolic and electrolyte toxicity	Increased gluconeogenesis and altered sodium, fluid, and potassium balance	Official safety information and RCTs (Sprung et al., 2008; Annane et al., 2018; Dailymed, 2026a; Keh et al., 2016; Loisa et al., 2007)	Hyperglycemia, hypernatremia, fluid retention, and insulin-treatment burden	No validated dose-duration threshold for contemporary low-dose regimens
Immune, infectious, and neuromuscular toxicity	Suppression of host defense and catabolic effects on skeletal muscle	RCT, label, and systematic/observational evidence (Sprung et al., 2008; Annane et al., 2018; Dailymed, 2026a; Yang et al., 2018)	Secondary infection, recurrent sepsis/shock, and ICU-acquired weakness	Absolute risks are regimen- and host-dependent; several estimates are imprecise

Evidence levels: clinical = randomized or physiological studies in patients with septic shock; translational = studies in critically ill patients or human experimental models; preclinical = molecular, cellular, or animal evidence. Official pharmacology information is used for drug properties and recognized adverse effects.

Interpretive boundary: no individual mechanism in this table constitutes a validated treatment-selection biomarker.

## 4 Clinical pharmacology and trial evidence

The clinical evidence for corticosteroids in sepsis is best understood as an evolution in drug, dose, exposure duration, target population, and therapeutic objective. The early high-dose strategy attempted rapid, broad immunosuppression; contemporary low-dose regimens instead aim to restore vascular responsiveness and accelerate liberation from vasopressors. Consequently, trials should not be classified simply as positive or negative. Their findings reflect different corticosteroid strategies applied to populations with different shock burdens, treatment windows, co-interventions, and outcome priorities.

### 4.1 Failure of high-dose, short-course regimens

The high-dose trials of the 1980s established the exposure boundary that continues to shape modern practice. In a 59-patient study, high-dose methylprednisolone or dexamethasone produced more 24-h shock reversal than control, but did not improve hospital survival (Sprung et al., 1984). A larger trial of high-dose methylprednisolone in severe sepsis and septic shock likewise showed no overall benefit. Among patients with a baseline serum creatinine concentration above 2 mg/dL, 14-day mortality was 59% with methylprednisolone and 29% with placebo, providing an important—although subgroup-based—harm signal (Bone et al., 1987). These findings do not imply that every corticosteroid exposure is harmful. Rather, they show that a short burst of intensive immunosuppression can generate transient physiological effects without durable benefit and may amplify organ-specific risk. This historical distinction is preserved in the 2024 Society of Critical Care Medicine focused update, which strongly recommends against regimens exceeding 400 mg/day hydrocortisone equivalent for fewer than 3 days in adults with septic shock (Chaudhuri et al., 2024a).

### 4.2 Low-dose corticosteroids: shock reversal, mortality, and organ support

The modern low-dose paradigm emerged from the 2002 trial by Annane and colleagues. Patients with vasopressor- and ventilator-dependent septic shock received hydrocortisone 50 mg every 6 hours plus fludrocortisone 50 μg daily for 7 days. Among corticotropin nonresponders, 28-day mortality was 53% with hydrocortisone plus fludrocortisone and 63% with placebo, and vasopressor withdrawal occurred earlier. In the overall population, however, 28-day mortality was 55% versus 61% and did not reach statistical significance (Annane et al., 2002). The trial therefore established a clinically influential combination strategy, but it did not validate corticotropin response as a durable prescribing biomarker.

CORTICUS broadened eligibility and tested hydrocortisone monotherapy in 499 patients. Hydrocortisone did not reduce 28-day mortality overall or according to corticotropin response, but shortened the median time to shock reversal from 5.8 to 3.3 days (Sprung et al., 2008). The trial also clarified that hemodynamic benefit is not synonymous with net clinical benefit. In its per-protocol safety population, hyperglycemia occurred in 85% versus 72% and hypernatremia in 29% versus 18% of patients receiving hydrocortisone and placebo, respectively. Superinfection and recurrent-shock events were numerically more frequent, although several infection-related estimates were imprecise (Sprung et al., 2008).

Two 2016 trials further defined the population and timing boundaries. HYPRESS tested hydrocortisone before shock had developed. Among adults with severe sepsis without septic shock, hydrocortisone did not reduce progression to shock by day 14 (21.2% vs. 22.9%) and increased the burden of hyperglycemia (Keh et al., 2016). The study therefore argues against prophylactic hydrocortisone treatment in unselected sepsis rather than against treatment of established vasopressor-dependent shock. VANISH enrolled patients early after shock onset in a 2-by-2 factorial comparison of vasopressin versus norepinephrine and hydrocortisone versus placebo. Hydrocortisone did not clearly improve mortality, shock reversal, kidney outcomes, or serious adverse events; 28-day mortality was 30.8% versus 27.5% (absolute difference 3.3 percentage points, 95% CI −5.5 to 12.1) (Gordon et al., 2016). Because the trial was primarily designed around vasopressor strategy and kidney failure-free days, its hydrocortisone comparison should be interpreted asan informative but not definitive test of early hydrocortisone initiation.

ADRENAL and APROCCHSS provide the most influential contemporary evidence, but they evaluated different treatment versions in different target populations. ADRENAL randomized mechanically ventilated patients with septic shock to a continuous infusion of hydrocortisone 200 mg/day or placebo. Hydrocortisone did not lower 90-day mortality (27.9% vs. 28.8%; OR 0.95, 95% CI 0.82–1.10; P = 0.50) but accelerated shock resolution (median 3 vs. 4 days; HR 1.32, 95% CI 1.23–1.41; P < 0.001) (Venkatesh et al., 2018). APROCCHSS tested hydrocortisone 50 mg every 6 hours plus oral fludrocortisone 50 μg daily for 7 days without tapering in a population with more severe shock. Ninety-day mortality was 43.0% with the combination and 49.1% with placebo (RR 0.88, 95% CI 0.78–0.99; P = 0.03); vasopressor-free days were also greater (median 17 vs. 15 days; P < 0.001), as were organ-failure-free days (14 vs. 12 days; P = 0.003) (Annane et al., 2018). At the class level, the 2024 Pitre meta-analysis of 45 randomized trials estimated a modest reduction in short-term mortality (RR 0.93, 95% CI 0.88–0.99) together with a more pronounced increase in 7-day shock reversal (RR 1.24, 95% CI 1.11–1.38) (Pitre et al., 2024). These pooled estimates describe an average corticosteroid-class effect across heterogeneous trials and should not be interpreted as evidence that hydrocortisone monotherapy, hydrocortisone plus fludrocortisone therapy, or historical high-dose regimens have equivalent benefit–harm profiles (Pitre et al., 2024). Taken together, these trials support a consistent hemodynamic effect from contemporary low-dose regimens while showing that mortality benefit is not uniform across treatment versions and populations.

### 4.3 Regimen heterogeneity: molecule, administration, duration, and withdrawal

Differences between ADRENAL and APROCCHSS should not be reduced to a simple conflict between trials. ADRENAL evaluated continuous hydrocortisone monotherapy in mechanically ventilated patients and allowed treatment to stop at intensive care unit discharge, whereas APROCCHSS evaluated a fixed-duration regimen of intermittent hydrocortisone plus enteral fludrocortisone in a higher-risk population. These differences simultaneously involve target population, drug composition, route, exposure profile, withdrawal strategy, and background risk. Cross-trial comparison cannot isolate the independent contribution of any one component (Venkatesh et al., 2018; Annane et al., 2018). Accordingly, evidence for a pooled corticosteroid-class effect should be separated from evidence for a specific treatment version: hydrocortisone monotherapy and hydrocortisone plus fludrocortisone are distinct regimens, and indirect cross-trial comparison cannot determine whether their mortality effects differ because of fludrocortisone, route, dose pattern, treatment duration, population severity, or other trial-level differences.

The incremental effect of fludrocortisone remains a particularly important pharmacological question. COIITSS directly compared hydrocortisone plus fludrocortisone with hydrocortisone alone and found no significant difference in in-hospital mortality (42.9% vs. 45.8%; RR 0.94, 95% CI 0.77-1.14; P = 0.50) or organ-support-free outcomes (COIITSS Study Investigators, 2010). The fludrocortisone comparison was secondary and not included in the sample-size calculation, and no insulin-by-fludrocortisone interaction was detected (P = 0.31); the insulin arm is therefore a co-randomized factor rather than conventional confounding. APROCCHSS supports the combination versus placebo, while FluDReSS found no faster shock resolution (Annane et al., 2018; Walsham et al., 2024). Direct randomized evidence is therefore neutral but imprecise, leaving the independent contribution of fludrocortisone unresolved.

Administration mode may modify peak–trough exposure and metabolic toxicity. In a 48-patient randomized study, continuous and intermittent hydrocortisone produced similar mean glucose concentrations, but intermittent boluses generated more hyperglycemic episodes and required more insulin adjustments (Loisa et al., 2007). Later small studies have not established a consistent clinical-outcome advantage for either approach. Glycemic variability may therefore serve as a pharmacodynamic and safety indicator, but it is not a validated surrogate for survival or overall benefit.

Duration and withdrawal strategies are similarly unsettled. CORTICUS tapered hydrocortisone after 5 days; VANISH used a staged reduction that could be accelerated after shock resolution; ADRENAL continued treatment for up to 7 days or until death or intensive care unit discharge; and APROCCHSS used a fixed 7-day course without tapering (Sprung et al., 2008; Venkatesh et al., 2018; Annane et al., 2018; Gordon et al., 2016). No head-to-head trial has established the superiority of fixed-duration treatment, response-guided discontinuation, tapering, or abrupt withdrawal. These trial protocols define tested treatment versions, not a universal bedside rule.

### 4.4 Contemporary guideline boundary

Current guidelines place corticosteroids within the treatment of established adult septic shock rather than unselected sepsis. The 2024 Society of Critical Care Medicine focused update conditionally suggests corticosteroids for adults with septic shock, based on low-certainty evidence, while strongly recommending against high-dose, short-duration therapy (Chaudhuri et al., 2024a). The 2026 Surviving Sepsis Campaign similarly suggests intravenous corticosteroids for adults with septic shock, again with low certainty of evidence (Prescott et al., 2026). The publicly available recommendation does not define a mandatory norepinephrine-equivalent dose at which treatment must begin.

These recommendations should be interpreted as an eligibility boundary rather than an individualized initiation rule. Corticosteroids do not replace adequate resuscitation, source-control assessment, antimicrobial therapy, or evaluation for hypovolemic, cardiogenic, hemorrhagic, or obstructive causes of persistent hypotension. Conversely, the absence of a fixed vasopressor threshold does not imply that every patient receiving a low dose of norepinephrine should receive hydrocortisone. Current guidance identifies a population in whom treatment may be considered; it does not identify who will derive net benefit.

### 4.5 What the trials establish—and what they do not

Three conclusions are comparatively robust. First, high-dose, short-course corticosteroid therapy should not be used as broad anti-inflammatory treatment for sepsis. Second, prophylactic hydrocortisone in severe sepsis without shock is not supported. Third, among adults with established septic shock, contemporary low-dose regimens most reproducibly accelerate shock reversal and vasopressor liberation (Chaudhuri et al., 2024a; Prescott et al., 2026; Sprung et al., 2008; Venkatesh et al., 2018; Sprung et al., 1984). Mortality benefit is regimen- and population-dependent: it was demonstrated for the hydrocortisone plus fludrocortisone strategy in APROCCHSS but not for hydrocortisone monotherapy in ADRENAL or CORTICUS.

The trials do not establish a universal vasopressor threshold, optimal initiation time, preferred administration mode, ideal duration, withdrawal strategy, or a precise incremental effect of fludrocortisone. COIITSS directly randomized fludrocortisone on a common hydrocortisone background, but the comparison was secondary and underpowered for modest effects; cross-trial differences among COIITSS, ADRENAL, and APROCCHSS cannot resolve the remaining uncertainty (Venkatesh et al., 2018; Annane et al., 2018; COIITSS Study Investigators, 2010). The trials also do not provide a reproducible individual responder rule. Randomized evidence therefore defines tested regimen-specific effects, while meta-analysis summarizes average class-level effects across trials; neither by itself provides a reproducible individual responder rule, while additional enrichment is required to identify patients with a more favorable individual benefit-harm profile. Table 3 summarizes these prescribing implications and limitations.

**TABLE 3:** Major randomized corticosteroid trials in sepsis and septic shock: populations, treatment versions, outcomes, and relevance to precision pharmacotherapy.

Study/year	Key eligibility and severity	Corticosteroid regimen	Duration/taper	Comparator
Part A. Trial populations and corticosteroid treatment versions				
Sprung 1984 (Sprung et al., 1984) (n = 59)	Established septic shock with persistent hypotension despite fluid resuscitation and evidence of hypoperfusion; most patients required vasopressors	Methylprednisolone 30 mg/kg IV or dexamethasone 6 mg/kg IV	Initial dose; repeated once after 4 h if shock persisted; no further study corticosteroid	No-corticosteroid control
Bone 1987 (Bone et al., 1987) (n = 382)	Severe sepsis or septic shock with infection, systemic manifestations, and organ dysfunction; treatment initiated early after meeting entry criteria	Methylprednisolone 30 mg/kg IV q6h	Four doses over 24 h; no taper	Placebo
Annane 2002 (Annane et al., 2002) (n = 300 randomized; 299 analyzed)	Catecholamine-dependent septic shock with organ dysfunction and mechanical ventilation; enrollment within 8 h of shock onset	HC 50 mg IV q6h + FC 50 μg enterally once daily	7 days; taper not specified	Matching HC and FC placebos
CORTICUS 2008 (Sprung et al., 2008) (n = 499)	Septic shock within 72 h; persistent hypotension or vasopressor requirement despite adequate fluid replacement, with sepsis-related organ dysfunction	HC 50 mg IV q6h	Days 1–5, followed by taper through day 11	Placebo
COIITSS 2010 (COIITSS Study Investigators, 2010) (n = 509)	Severe sepsis with SOFA ≥8 and vasopressor dependence; all patients already receiving HC for septic shock	Background HC 50 mg IV q6h ± FC 50 μg enterally once daily	7 days; taper not specified	HC alone; insulin strategy co-randomized in a 2 × 2 factorial design
HYPRESS 2016 (Keh et al., 2016) (n = 380 randomized; 353 ITT)	Severe sepsis with organ dysfunction but without septic shock; severe sepsis present ≤48 h	HC 50 mg IV bolus followed by continuous IV infusion	200 mg/day days 1–5 → 100 mg/day days 6–7 → 50 mg/day days 8–9 → 25 mg/day days 10–11	Placebo
VANISH 2016 (Gordon et al., 2016) (n = 409)	Septic shock requiring vasopressors despite adequate fluid resuscitation; enrollment within 6 h of vasopressor initiation	HC 50 mg IV q6h as conditional second study drug	5 days q6h → 3 days q12 h → 3 days once daily; faster weaning permitted after shock resolution	Placebo within vasopressin/norepinephrine 2 × 2 factorial design
ADRENAL 2018 (Venkatesh et al., 2018) (n = 3,800 randomized)	Mechanically ventilated adults with septic shock receiving vasopressors/inotropes for ≥4 h; eligibility present ≤24 h	HC 200 mg/day by continuous IV infusion	Maximum 7 days or until ICU discharge or death; no taper	Placebo
APROCCHSS 2018 (Annane et al., 2018) (n = 1,241)	Severe septic shock <24 h with persistent high-dose vasopressor requirement and multiple organ dysfunction	HC 50 mg IV q6h + FC 50 μg enterally once daily	7 days without tapering	Matching placebos; initially embedded in a 2 × 2 factorial design
FluDReSS 2024 (Walsham et al., 2024) (n = 153)	Mechanically ventilated, vasopressor-dependent septic shock already treated with HC 200 mg/day; eligibility ≤24 h	Background HC 200 mg/day + FC 0, 50, 100, or 200 μg/day enterally	Maximum 7 days or until ICU discharge, HC cessation, or death	HC alone vs. HC + three FC dose regimens

Study/year (sample size)	Mortality outcome	Shock/hemodynamic outcome	Adverse events/safety	Major methodological limitations	Relevance to precision treatment
Part B. Clinical outcomes and interpretive boundaries					
Sprung 1984 (Sprung et al., 1984)	In-hospital mortality: methylprednisolone 76%, dexamethasone 77%, control 69%; no significant benefit	Shock reversal 57%, 59%, and 38%, respectively; early reversal favored corticosteroids but was not sustained	Superinfection and hyperglycemia were reported; estimates were imprecise	Very small trial; incomplete blinding; historical definitions and supportive care	Historical high-dose evidence; transient hemodynamic improvement did not establish survival benefit or a contemporary responder rule
Bone 1987 (Bone et al., 1987)	14-day mortality: 32% vs. 25%, P = 0.06	No significant benefit in overall shock prevention or reversal	Overall secondary-infection incidence was similar, but infection-attributed mortality was higher with methylprednisolone	Historical definitions and very high-dose regimen; subgroup findings were not designed as predictive interactions	Supports rejection of high-dose regimens; renal subgroup signals should not be used for treatment selection
Annane 2002 (Annane et al., 2002)	Nonresponders: 28-day mortality 53% vs. 63%; HR 0.67 (95% CI 0.47–0.95), P = 0.02. Overall: 55% vs. 61%, not significant	In nonresponders, vasopressor withdrawal was faster: median 7 vs. 10 days; HR 1.91 (95% CI 1.29–2.84)	Overall adverse-event rates were similar	Primary hypothesis centered on corticotropin nonresponders; sample-size assumptions differed substantially from observed event rates	Supports the HC + FC regimen in severe shock, but does not independently establish FC benefit or validate ACTH-guided selection
CORTICUS 2008 (Sprung et al., 2008)	28-day mortality 34.3% vs. 31.5%, P = 0.51; no benefit according to corticotropin response	Shock-reversal proportion was similar, but median time to reversal was shorter with HC: 3.3 vs. 5.8 days	More superinfection/new sepsis, hyperglycemia, and hypernatremia with HC	Underpowered: ∼500 enrolled vs. 800 planned; some open-label corticosteroid use	Demonstrates hemodynamic benefit without survival benefit; corticotropin response did not provide a reproducible selection rule
COIITSS 2010 (COIITSS Study Investigators, 2010)	HC + FC vs. HC: in-hospital mortality 42.9% vs. 45.8%; RR 0.94 (95% CI 0.77–1.14), P = 0.50	No significant difference in vasopressor-free days, ventilation-free days, or SOFA recovery	Superinfection was more frequent with FC, particularly urinary infection	FC comparison was secondary, not included in the sample-size calculation, and open label	Direct HC + FC vs. HC comparison, but insufficiently powered to exclude a modest incremental FC effect
HYPRESS 2016 (Keh et al., 2016)	28-day mortality 8.8% vs. 8.2%, P = 0.86; no mortality benefit through 180 days	Septic shock by day 14: 21.2% vs. 22.9%, P = 0.70	Hyperglycemia increased; no significant differences in secondary infection, weakness, or hypernatremia	Consent may have excluded rapidly deteriorating patients; shock incidence was lower than assumed; several secondary analyses were exploratory	Defines a pre-shock boundary: does not support prophylactic HC before established septic shock
VANISH 2016 (Gordon et al., 2016)	HC vs. placebo: 28-day mortality 30.8% vs. 27.5%; absolute difference 3.3% (95% CI −5.5 to 12.1); interaction with vasopressin P = 0.98	HC reduced vasopressin requirements; no clear hydrocortisone effect on kidney outcomes or mortality	No significant difference in serious adverse events between HC and placebo	HC/placebo was a conditional second intervention; many patients never received study drug 2, limiting power for hydrocortisone and interaction effects	Early-shock factorial evidence, but not a definitive stand-alone hydrocortisone trial; timing alone did not establish individualized benefit
ADRENAL 2018 (Venkatesh et al., 2018)	90-day mortality 27.9% vs. 28.8%; OR 0.95 (95% CI 0.82–1.10), P = 0.50	Faster shock resolution: median 3 vs. 4 days; HR 1.32 (95% CI 1.23–1.41), P < 0.001	Trial-related AEs 1.1% vs. 0.3%; no difference in new bacteremia/fungemia	AE ascertainment was restricted to clinician-judged treatment-related events; not all infections or long-term neuromuscular outcomes were assessed	Strong evidence that HC accelerates shock resolution without reducing 90-day mortality; no validated responder subgroup emerged
APROCCHSS 2018 (Annane et al., 2018)	90-day mortality 43.0% vs. 49.1%; RR 0.88 (95% CI 0.78–0.99), P = 0.03	Vasopressor-free days: 17 vs. 15, P < 0.001; organ-failure-free days: 14 vs. 12, P = 0.003	Hyperglycemia increased; serious AEs, bleeding, and superinfection were not significantly increased	Original factorial design changed after drotrecogin alfa withdrawal; secondary outcomes were not multiplicity-adjusted; HC + FC was tested as a fixed combination	Establishes regimen-specific HC + FC benefit vs. placebo; does not prove a generic corticosteroid class effect or isolate FC contribution
FluDReSS 2024 (Walsham et al., 2024)	28-day mortality: HC alone 23.7%; FC 50, 100, and 200 μg/day: 16.7%, 11.1%, and 10.8%; not powered for mortality	Median shock-resolution time ≈3 days in all groups; no significant dose-response effect	No significant differences in prespecified electrolyte, fluid-balance, or infection outcomes	Phase II; prematurely terminated and underpowered; open label; vasopressor weaning not protocolized; primary outcome not patient-centered	Provides dose-finding and PK evidence; no demonstrated clinical dose-response and insufficient evidence for FC dose-guided prescribing

Abbreviations: CI, confidence interval; FC, fludrocortisone; HC, hydrocortisone; HR, hazard ratio; ICU, intensive care unit; ITT, intention-to-treat; IV, intravenous; OR, odds ratio; RR, relative risk; SOFA, Sequential Organ Failure Assessment. Effect estimates are reported according to the original trial publications and were not re-standardized across studies. Free-day outcomes are reported according to the definitions used in the original trials and should be interpreted alongside mortality and the prespecified handling of death. “Precision-treatment relevance” reflects the authors’ interpretation of what each trial can and cannot establish for individualized corticosteroid treatment.

## 5 Heterogeneous treatment effects and biomarker enrichment

Randomized trials estimate the average effect of a corticosteroid regimen in a defined population, but the same average can conceal clinically important distributions of benefit, nonresponse, and harm. Precision pharmacotherapy therefore requires more than identifying patients at high risk. A pretreatment characteristic becomes a candidate predictive marker only when it modifies the effect of corticosteroid exposure relative to an appropriate comparator. This distinction separates prognostic enrichment, which selects patients with a higher event probability, from predictive enrichment, which seeks differential relative treatment effects. Higher baseline risk may increase trial efficiency and absolute benefit even when the relative effect is constant; it does not establish greater biological responsiveness. Formal treatment-by-marker interaction is consequently more informative than comparing outcomes across separate prognostic strata, because only interaction analyses can evaluate whether treatment effects differ according to a candidate modifier (Iwashyna et al., 2015).

### 5.1 Prognostic stratification versus predictive enrichment

A fundamental distinction in precision medicine is the separation between prognostic stratification and predictive enrichment. Prognostic markers identify patients with different baseline risks of clinical outcomes irrespective of treatment assignment, whereas predictive markers identify characteristics associated with differences in treatment effect across alternative therapeutic strategies. Accordingly, a marker associated with greater disease severity, mortality risk, or biological disturbance should not be considered a treatment-selection biomarker unless treatment-effect modification has been demonstrated (Stanski and Wong, 2020).

Statistically, treatment-effect modification is evaluated by directly comparing treatment effects across levels of the candidate marker rather than by comparing the statistical significance of treatment effects within separate subgroups. In a regression framework, this is commonly represented by terms for treatment, marker status, and their treatment-by-marker interaction; the interaction term tests whether the treatment effect differs according to marker status on the prespecified effect scale. Importantly, a statistically significant treatment effect in one subgroup and a nonsignificant effect in another does not by itself establish interaction; the difference between the treatment-effect estimates must itself be evaluated (Iwashyna et al., 2015; Stanski and Wong, 2020; Altman and Bland, 2003). Because interaction is scale-dependent, both relative and absolute treatment effects should be reported when clinically relevant.

A simple hypothetical example illustrates the distinction. Suppose 28-day mortality without corticosteroids is 40% in a high-risk marker-positive group and 10% in a lower-risk marker-negative group. If corticosteroid treatment reduces mortality to 32% and 8%, respectively, the relative risk is 0.80 in both groups, although the absolute risk reduction is larger in the high-risk group (8 versus 2 percentage points). The larger absolute benefit therefore reflects higher baseline risk rather than evidence that the marker predicts greater relative corticosteroid responsiveness. By contrast, if treatment reduced mortality from 40% to 24% in the marker-positive group but from 10% to 9% in the marker-negative group, the corresponding relative risks would be 0.60 and 0.90; this pattern would suggest potential treatment-effect modification and should be evaluated with a formal interaction test and its uncertainty. These numerical examples are illustrative only and are not derived from corticosteroid trials.

This distinction is particularly important in sepsis, where high disease severity may improve trial efficiency and increase the potential absolute benefit of an effective therapy without identifying patients with a different biological response to corticosteroids. Prognostic enrichment therefore answers who is at higher baseline risk, whereas predictive enrichment asks whether the benefit–harm contrast of a defined corticosteroid strategy differs according to a pretreatment characteristic. Only the latter can ultimately support individualized treatment selection after adequate replication and prospective validation (Stanski and Wong, 2020).

### 5.2 Clinical severity and prognostic enrichment

Clinical severity represents the most established form of prognostic enrichment in septic shock, but severity alone does not establish differential corticosteroid responsiveness.

Corticosteroid evidence illustrates the danger of treating severity as a predictive biomarker. In a calibration analysis of 1,089 patients from 10 randomized trials, L’Abbé and pooled calibration plots did not demonstrate a significant interaction between illness severity and corticosteroid effectiveness. Three trials had higher-than-predicted mortality in their placebo groups; after these studies were excluded, the pooled mortality estimate moved from an odds ratio of 0.85 toward the null (OR 0.97, 95% CI 0.71–1.33) (Ho and Tan, 2011). These findings suggest that an apparent greater benefit among higher-risk populations may partly reflect baseline-risk calibration rather than reproducible patient-level treatment-effect modification. Pediatric data provide a related caution. PERSEVERE-based mortality-risk strata did not identify a subgroup with improved outcome after corticosteroid exposure (Atkinson et al., 2014). In a subsequent secondary analysis combining prognostic and transcriptomic enrichment, corticosteroid exposure among endotype B children at intermediate-to-high PERSEVERE-predicted risk was associated with a lower risk of complicated course (RR 0.09, 95% CI 0.01–0.54) (Wong et al., 2016). However, corticosteroid treatment was not randomized in these pediatric analyses, and the investigators explicitly called for prospective validation. The result should therefore be considered hypothesis-generating rather than evidence of a validated predictive treatment interaction.

Accordingly, severity scores and prognostic models can improve eligibility definition, trial efficiency, and interpretation of absolute treatment benefit, but they should not be presented as corticosteroid-response classifiers. A high-risk patient may have more absolute outcome events available to prevent without having a different relative biological response to corticosteroids. This distinction is particularly important for dynamic phenotypes, because trajectory-based severity patterns may improve prognostic classification without necessarily demonstrating treatment-effect modification.

### 5.3 Transcriptomic endotypes and corticosteroid-response evidence

Transcriptomic endotyping aims to identify biologically distinct host-response states that may be relevant to corticosteroid responsiveness, including variation in adaptive immune activity, innate inflammatory signaling, glucocorticoid-receptor-related programs, and cellular stress responses (Wong et al., 2016; Scicluna et al., 2017). The major classification frameworks relevant to corticosteroid-response research—including adult SRS1/SRS2, pediatric endotype A/B, IA-P/IN-P, and the more recent consensus transcriptomic subtypes (CTS1–CTS3)—were developed in different populations using different gene sets, platforms, and classification procedures (Antcliffe et al., 2019; Wong et al., 2016; Venkatesh et al., 2025). These frameworks may capture partially overlapping host-response biology, but they are not interchangeable, and directional similarity across classifiers should not be interpreted as formal external validation (Sciclun et al., 2025).

In the VANISH transcriptomic secondary analysis, pretreatment whole-blood RNA was available from 176 adults with septic shock and was classified using the SRS framework (Antcliffe et al., 2019). Hydrocortisone or placebo was administered as study drug 2 only after the maximum infusion rate of the initially assigned vasopressor had been reached; consequently, the hydrocortisone-by-SRS analysis included 117 patients who actually received randomized study drug 2 rather than all 176 transcriptomically classified participants (Antcliffe et al., 2019). The published classification was later corrected after 39 of 176 participants (22%) were found to have been misclassified. In the corrected data, 82 patients were assigned to SRS1 and 94 to SRS2. The hydrocortisone-by-SRS interaction for 28-day mortality remained statistically significant (P = 0.02), and hydrocortisone was associated with higher 28-day mortality in SRS2 (OR 4.6, 95% CI 1.5–14.4) (Antcliffe et al., 2022). This signal challenges a simple model in which greater baseline immunosuppression necessarily predicts greater hydrocortisone-related harm. However, this was a secondary, *post hoc* transcriptomic analysis of a subset of VANISH participants; the classification required correction for substantial misclassification, subgroup estimates remained imprecise, and no prospective SRS-guided trial has tested treatment selection based on this classifier. Accordingly, SRS classification should be regarded as a candidate treatment-effect modifier rather than a validated individual hydrocortisone-selection tool (Antcliffe et al., 2019).

The pediatric endotype A/B framework was developed independently. Endotype A is characterized by repression of adaptive-immunity and glucocorticoid-receptor-signaling programs and a higher baseline mortality risk. Corticosteroid exposure in endotype A was associated with increased mortality in an initial clinically feasible classifier and a simplified follow-up classifier (Wong et al., 2015; Wong et al., 2017). Because corticosteroid prescription in these pediatric studies was not randomized, these associations remain vulnerable to confounding by indication and should not be interpreted as definitive causal treatment effects. Longitudinal analysis showed that 77 of 243 children (32%) assigned to endotype B on day 1 transitioned to endotype A by day 3, demonstrating that host-response states can change during the treatment window; corticosteroid exposure was also associated with increased mortality among patients who persisted as endotype A (Wong et al., 2018). Exploratory application of the pediatric classifier to 97 adults in VANISH showed 28-day mortality of 46% with hydrocortisone versus 22% with placebo among endotype A patients (P = 0.105), whereas mortality in endotype B was 19% versus 22%, respectively (Wong et al., 2021). These findings remain exploratory for different reasons: the pediatric associations are vulnerable to confounding by indication because treatment was nonrandomized, whereas the adult VANISH analysis was limited by small sample size, wide uncertainty, and *post hoc* transport of a pediatric classifier into an adult population.

The 2025 ADRENAL transcriptomic substudy provides a more recent adult evaluation. In 540 randomized participants, the primary analysis did not demonstrate heterogeneity in the effect of hydrocortisone on 28-day mortality between the immune adaptive-prevalent (IA-P) and immune innate-prevalent (IN-P) signatures (ratio of odds ratios [ROR] 0.97, 95% CrI 0.39–2.49) (Venkatesh et al., 2025). A prespecified severe-shock subgroup was similarly inconclusive (ROR 0.61, 95% CrI 0.12–2.91). In contrast, within the prespecified pulmonary-sepsis subgroup, a differential treatment effect by endotype was observed (ROR 0.18, 95% CrI 0.03–0.97), driven by increased mortality with hydrocortisone in IA-P patients (OR 5.55, 95% CrI 1.81–21.12), whereas the corresponding estimate in IN-P was 1.01 (95% CrI 0.29–3.38) (Sciclun et al., 2025). Because the overall endotype analysis was negative and this finding arose in a smaller subgroup with wide uncertainty, it should remain exploratory pending independent prospective replication. An earlier prespecified ADRENAL candidate-gene substudy found no association between candidate adrenocortical gene-expression levels and 28-day mortality, 90-day mortality, or overall time to shock reversal (Cohen et al., 2021). However, treatment-by-gene interactions for time to shock resolution were observed for GLCCI1 (P for interaction = 0.008) and HSD11B1 (P for interaction = 0.014). Nominal treatment interactions for mortality involving CRH and LINC02210-CRHR1 were not robust to sensitivity analysis, and the study did not apply a correction for multiple comparisons (Cohen et al., 2021). These findings therefore remain exploratory and do not establish a validated molecular treatment-selection rule.

Against this background of partially overlapping but noninterchangeable transcriptomic classifications, Scicluna and colleagues recently developed a consensus blood transcriptomic framework that aligned previously reported sepsis transcriptomic classifications into three consensus transcriptomic subtypes (CTS1–CTS3) (Sciclun et al., 2025). The framework was developed using the MARS and GAinS cohorts and subsequently evaluated in independent cohorts, including the VANISH randomized trial, supporting the reproducibility of CTS assignment across distinct datasets (Sciclun et al., 2025). In the propensity-score-matched MARS analysis, CTS2 was associated with higher 28-day mortality, but the CTS2-by-corticosteroid interaction did not significantly differ from the CTS1 reference (OR 0.56, 95% CI 0.16–1.94; P = 0.363); an interaction favoring CTS3 relative to CTS1 was observed (OR 0.20, 95% CI 0.06–0.66; P = 0.009), although this analysis remained observational and nonrandomized (Sciclun et al., 2025). In the VANISH reanalysis, hydrocortisone-by-CTS interaction terms were not statistically significant, whereas CTS2 itself remained associated with higher 28-day mortality (OR 5.76, 95% CI 1.31–25.4; P = 0.021) (Sciclun et al., 2025). Thus, the high mortality observed among corticosteroid-treated CTS2 patients should not be interpreted as proof of a CTS2-specific corticosteroid treatment effect. Thus, the CTS findings strengthen the rationale for prospective transcriptomic enrichment in corticosteroid trials but do not alter the current conclusion that no transcriptomic subtype has been validated as a routine corticosteroid-selection or exclusion criterion.

### 5.4 A framework for predictive enrichment and treatment-effect interaction

Building on the statistical distinction above, predictive enrichment requires candidate biomarkers, phenotypes, or trajectories to undergo prespecified and replicated treatment-effect interaction testing before they can inform treatment selection (Stanski and Wong, 2020).

We use the FDA–NIH BEST terminology to distinguish four roles for candidate markers (Table 4) (U. S. Food and Drug Adminstration, 2021; FDA-NIH Biomar ker Working Group, 2016). Prognostic markers describe baseline risk, whereas predictive markers require evidence of treatment-effect modification. Pharmacodynamic measures characterize biological or physiological response after treatment, and safety indicators capture susceptibility to or emergence of toxicity.

**TABLE 4:** Four-domain enrichment framework for precision corticosteroid research.

Enrichment domain	Core question	Candidate variables or indicators	Measurement timing	What it can/cannot support	Current readiness
Prognostic enrichment	Who is at higher baseline risk?	SOFA, lactate, vasopressor burden, baseline organ function, PERSEVERE risk	Before treatment	Supports risk stratification, trial efficiency, and estimation of absolute risk/benefit. Does not establish relative corticosteroid-effect modification without a treatment-by-marker interaction	Moderate for prognosis; limited corticosteroid specificity
Predictive enrichment	Who benefits or is harmed relative to a comparator?	SRS1/SRS2; pediatric Endotype A/B; IA-P/IN-P; validated treatment-interaction signatures	Before treatment; result must return within the decision window	Can support prospective enrichment or treatment selection only after replicated interaction testing. Cannot be inferred from prognosis alone or significance in a single subgroup	Low; research enrichment, not routine prescribing
Pharmacodynamic/response indicators	Did the drug produce an expected biological or physiological response?	Norepinephrine-equivalent dose decline; MAP response; lactate change; inflammatory or immune-response signals	After treatment; may be serial	Supports dose-response and early response characterization. Does not by itself prove efficacy, net benefit, pretreatment responsiveness, or surrogate validity	Low to moderate as candidate response indicators; no qualified corticosteroid-specific pharmacodynamic biomarker
Safety/toxicity indicators	Who is vulnerable, and is treatment-related harm emerging?	Pretreatment diabetes/sodium status; post-treatment glucose/sodium; infection signals; weakness or delirium	Before treatment for susceptibility; after treatment for monitoring	Supports toxicity surveillance and benefit-harm assessment. Risk factors, laboratory abnormalities, and adverse outcomes are not automatically qualified safety biomarkers	Moderate for routine safety monitoring; low for precision treatment selection

Framework based on FDA–NIH BEST, terminology and adapted by the authors to corticosteroid research in septic shock. Candidate examples do not necessarily meet formal regulatory biomarker qualification.

Abbreviations: BEST, biomarkers, EndpointS, and other Tools; IA-P, immune adaptive-prevalent; IN-P, immune innate-prevalent; MAP, mean arterial pressure; SRS, sepsis response signature.

(1) This is an author-proposed framework, not a validated prescribing tool. (2) Clinical outcomes such as death, shock reversal, and kidney replacement therapy are not biomarkers. (3) Corrected VANISH, estimates are used for SRS, results.

In randomized clinical trials, this requires prespecified or appropriately interpreted interaction analyses comparing treatment effects across biologically or clinically defined subgroups. Associations between a biomarker and poor outcome in untreated populations, although clinically meaningful, cannot alone establish predictive utility (Stanski and Wong, 2020).

For corticosteroid therapy in septic shock, this distinction is particularly relevant because large randomized controlled trials have demonstrated heterogeneous effects across populations despite similar treatment indications. Trials including CORTICUS, ADRENAL, APROCCHSS, VANISH, and COIITSS have provided important evidence regarding average treatment effects, but they have not established a universally applicable biomarker or phenotype for selecting corticosteroid responders (Sprung et al., 2008; Venkatesh et al., 2018; Annane et al., 2018; COIITSS Study Investigators, 2010; Gordon et al., 2016). Therefore, the next step in corticosteroid precision therapy is not simply identifying patients at higher risk, but determining whether specific biological or clinical characteristics modify corticosteroid-associated benefit or harm.

Timing is central to this distinction. Post-hydrocortisone changes in norepinephrine dose, MAP, or lactate may characterize response but cannot serve as pretreatment predictors. Likewise, death, shock reversal, KRT, infection, and ICU-acquired weakness are clinical outcomes rather than biomarkers.

### 5.5 Readiness for clinical translation

A candidate enrichment assay must demonstrate analytic validity, clinical validity, and clinical utility. It must classify reliably, reproduce the intended treatment interaction, and show that using the result improves decisions or reduces harm. Timing is equally important: a predictive result must be derived before corticosteroid exposure and returned within the clinical decision window. Most transcriptomic studies used stored specimens and offline classification, and external replication has often changed the classifier, threshold, specimen, or population. None of the current SRS, endotype A/B, or IA-P/IN-P strategies has completed this pathway.

The appropriate current role of biomarker enrichment is therefore prospective trial design and safety-focused hypothesis testing. Future studies should prespecify the assay, threshold, time zero, treatment version, interaction hypothesis, and multidimensional outcomes, and should replicate the same rule externally before clinical use. Until such evidence exists, clinical severity and transcriptomic endotypes should not be used as routine indications or contraindications; they remain complementary research layers that motivate the temporal evaluation developed in the next section.

## 6 Operationalizing trajectory-guided enrichment and causal evaluation

Baseline severity scores and transcriptomic endotypes cannot fully represent the direction of an evolving septic-shock course. Because vasopressor requirements, arterial pressure, lactate, organ dysfunction, resuscitation, and source control change together, precision pharmacotherapy requires strict separation of pretreatment information, treatment assignment, and post-exposure response. Longitudinal trajectories can provide phenotypic information that is unavailable from a single baseline measurement, but trajectory membership alone does not establish differential corticosteroid responsiveness (Stanski and Wong, 2020). Here, trajectory-guided enrichment refers to the use of a prespecified longitudinal phenotype—or a trajectory class predicted exclusively from information available before treatment—as a candidate treatment-effect modifier, followed by explicit evaluation of differential corticosteroid effects within a temporally aligned causal design (Rajendran et al., 2025; Hernán and Robins, 2016).

### 6.1 Why static baseline classification is insufficient

A single baseline value may conceal markedly different subsequent courses. In a multicohort study, patients with a rapidly worsening 72-h Sequential Organ Failure Assessment trajectory entered intensive care with a lower mean baseline score than rapidly improving patients, yet hospital mortality was 28.3% versus 5.5%. Early models predicted later trajectory membership with accuracies of 0.78 at 6 h and 0.87 at 24 h (Xu et al., 2022). Thus, disease direction adds information beyond initial severity, but a predicted future trajectory must still be distinguished from the observed trajectory on which its label was based.

Lactate, cortisol, and hemodynamic trajectories provide similar dynamic prognostic information (Ma et al., 2026; Shen et al., 2026; Leng et al., 2024). However, these trajectories are treatment-coupled clinical phenotypes: fluid therapy, vasopressor titration, ventilation, source control, and corticosteroid exposure can all alter the measured course. They may therefore generate treatment-effect hypotheses, but they do not become hydrocortisone-response markers without a prespecified treatment-by-trajectory analysis using information available before exposure. Observed trajectories and predicted trajectories therefore have different roles in precision-treatment research. An observed 48–72-h trajectory may be useful for phenotype discovery or prognosis, whereas a trajectory intended for treatment selection must be assigned or predicted from information available by the corticosteroid decision time (Rajendran et al., 2025; Xu et al., 2022). This distinction prevents a future clinical course—potentially already modified by corticosteroids, vasopressors, fluids, source control, or other interventions—from being retrospectively treated as a baseline predictor of treatment benefit (Hernán and Robins, 2016).

### 6.2 Operationalizing a trajectory-guided enrichment framework

Feature engineering should likewise be prespecified, including the sampling and aggregation interval, handling of irregularly measured or missing observations, and whether absolute level, slope, variability, cumulative burden, or other longitudinal summaries enter the trajectory model (Rajendran et al., 2025; Nagin et al., 2024). A trajectory-guided enrichment strategy should prespecify at least four elements: the longitudinal domain to be measured, the observation window relative to corticosteroid time zero, the method used to construct or predict the trajectory, and the treatment-effect question to which the trajectory will be applied (Hernán and Robins, 2016; Nagin et al., 2024). Clinically plausible domains in septic shock include hemodynamic evolution (for example, mean arterial pressure and norepinephrine-equivalent dose), organ-dysfunction evolution (SOFA score and individual organ components), and metabolic evolution (particularly serial lactate). Serial immune or transcriptomic measurements may also define biological state transitions, but a single baseline transcriptomic subtype remains a static enrichment feature rather than a trajectory (Stanski and Wong, 2020; Xu et al., 2022). The modeling approach should match the clinical and statistical question. Group-based or latent-class trajectory approaches summarize recurrent longitudinal patterns into interpretable subgroups; joint longitudinal–time-to-event models explicitly link repeated measurements with subsequent event risk; and clustering or machine-learning approaches can accommodate higher-dimensional or multidomain trajectories (Rajendran et al., 2025; Xu et al., 2022; Nagin et al., 2024; Rizopoulos, 2011). These approaches are not interchangeable: the number and stability of latent classes, measurement density, missingness, smoothing or functional-form assumptions, classification uncertainty, interpretability, and external reproducibility should be evaluated before a trajectory is carried forward as an enrichment variable; where membership is probabilistic, uncertainty in trajectory assignment should not be ignored in downstream treatment-effect analyses (Nagin et al., 2024). Most importantly, the observation window determines the role of the trajectory. If the complete trajectory is observed only after corticosteroid initiation, it can characterize pharmacodynamic response, mediation, prognosis, or toxicity, but it cannot validly serve as a pretreatment predictive enrichment variable (Stanski and Wong, 2020; Hernán and Robins, 2016). Table 5 summarizes candidate trajectory domains and explicitly separates phenotype-discovery windows from the information permissible for real-time pretreatment assignment.

**TABLE 5:** Candidate trajectory-guided enrichment framework for precision corticosteroid research in septic shock.

Trajectory domain or analytical application	Candidate longitudinal variables	Example discovery window/pretreatment-use constraint	Potential modeling approaches g	Clinical rationale	Representative evidence	Current status
Hemodynamic trajectory	MAP, vasopressor dose, norepinephrine-equivalent dose, vasopressor requirement pattern	Hours to 72 h may be used for phenotype discovery; for treatment selection, only repeated measurements obtained before corticosteroid time zero may contribute to assignment	GBTM, latent-class trajectory models, clustering	Captures persistence or resolution of vasoplegia beyond a single vasopressor value	Hemodynamic trajectory studies (Shen et al., 2026)	Exploratory; no validated corticosteroid-response trajectory
Organ-dysfunction trajectory	SOFA and organ-specific components	The first 72 h may be used to define discovery phenotypes; real-time enrichment requires prediction of future trajectory membership using only features available before corticosteroid time zero	GBTM/latent-class models, clustering; joint longitudinal–time-to-event models for dynamic outcome association	Captures direction of multisystem evolution beyond baseline severity	Xu et al. (Xu et al., 2022); Rajendran et al. (Rajendran et al., 2025)	Has direct Corticosteroid-specific proof-of-concept evidence; not validated for treatment selection
Metabolic trajectory	Lactate concentration, change and persistence	Serial early measurements; values after corticosteroid initiation belong to response rather than pretreatment enrichment	GBTM, longitudinal regression/spline models, clustering	May characterize persistence or resolution of metabolic stress	Lactate trajectory studies (Ma et al., 2026)	Mainly prognostic; predictive corticosteroid value unknown
Immune/biological trajectory	Serial transcriptomic states or inflammatory/immune biomarkers	Requires serial sampling; a single baseline SRS/CTS/endotype is not a trajectory	Longitudinal molecular clustering, latent-state models, ML	May capture transitions in host-response state	SRS/endotype studies (Venkatesh et al., 2025; Antcliffe et al., 2022; Wong et al., 2015; Wong et al., 2017; Wong et al., 2018; Wong et al., 2021; Cohen et al., 2021); consensus CTS framework (Sciclun et al., 2025)	Highly exploratory; no validated serial companion diagnostic
Composite trajectory phenotype	Multidomain physiology, organ dysfunction and biological features	All features used for treatment selection must be available before treatment time zero	Multimodal clustering, representation learning, dynamic ML	May capture interactions among evolving organ systems	Dynamic sepsis trajectory and phenotyping studies (Xu et al., 2022; Ma et al., 2026; Shen et al., 2026; Leng et al., 2024)	Hypothesis-generating; major transportability and interpretability requirements
Treatment-effect application	Predicted trajectory or trajectory-defined subgroup as candidate modifier	Subgroup assignment must occur before treatment comparison begins	Trajectory prediction + interaction analysis/TTE	Tests whether treatment effects differ across dynamically defined states	Rajendran et al. (Rajendran et al., 2025)	Proof-of-concept; not a validated prescribing strategy

Interpretive Discovery windows describe how candidate phenotypes may be derived; they do not define variables that are permissible for real-time treatment selection. For predictive enrichment, all information used to assign or predict subgroup membership must be available before corticosteroid time zero.

Abbreviations: CTS, consensus transcriptomic subtype; GBTM, group-based trajectory modeling; MAP, mean arterial pressure; ML, machine learning; SOFA, sequential organ failure assessment; SRS, sepsis response signature; TTE, target trial emulation.

### 6.3 Pre-treatment enrichment versus post-treatment response

A defensible temporal architecture separates three stages. The pre-exposure feature window contains variables used for eligibility, predictive enrichment, and baseline confounding control, such as prior corticosteroid use, norepinephrine-equivalent dose and slope, mean arterial pressure, lactate trend, organ dysfunction, infection source, and initial resuscitation. The treatment window defines an implementable strategy, for example, initiation of intravenous hydrocortisone at 200 mg/day within 6 h versus no initiation within the same window. The 6-h window is a research specification, not a validated clinical threshold. For trajectory-guided enrichment, the complete feature window used to assign the candidate subgroup must end before strategy assignment; otherwise, treatment-modified measurements can leak into the subgroup definition (Hernán and Robins, 2016; Hernán et al., 2025).

Post-exposure changes in vasopressor dose, arterial pressure, lactate, glucose, sodium, and organ function belong to pharmacodynamic, safety, mediation, or longitudinal-confounding analyses; they should not be recycled into a pretreatment benefit model. The same variable may serve different roles before and after time zero, but each role must be declared in advance. Hour-level studies should also distinguish actual drug administration from order, verification, dispensing, and documentation timestamps.

### 6.4 Target trial emulation for corticosteroid strategies

Target trial emulation is a design framework, not a synonym for propensity-score matching, inverse-probability weighting, or a time-dependent Cox model. The protocol should specify eligibility, treatment strategies, time zero, follow-up, outcomes, estimand, and analysis before estimation begins (Hernán and Robins, 2016; Cashin et al., 2025). For corticosteroid initiation, eligibility, strategy assignment, and follow-up should be aligned at the first clinically meaningful point when persistent vasopressor-dependent septic shock is present and both initiation and temporary deferral remain plausible. Target trial emulation does not itself identify a corticosteroid responder; trajectory-defined heterogeneity remains a separate treatment-effect-modification hypothesis that must be prespecified and evaluated explicitly (Hernán et al., 2025). Accordingly, trajectory-specific treatment effects should be reported with stratum-specific uncertainty and evaluated using a prespecified treatment-by-trajectory interaction or between-stratum treatment-effect contrast on a clinically relevant effect scale; statistical significance in one trajectory group and non-significance in another does not establish treatment-effect heterogeneity (Stanski and Wong, 2020; Altman and Bland, 2003).

When initiation is permitted during a grace period, patients may initially be compatible with both strategies. Clone-censor-weight or sequentially nested trial approaches can preserve this comparison by censoring strategy deviations and accounting for informative selection (Robins and Finkelstein, 2000; Cain et al., 2010). The estimand should match the question: an observational analogue of initial assignment differs from a per-protocol effect that also addresses adherence and later deviations. Absolute mortality risk, shock-reversal probability, and organ-support-free time are generally more interpretable for net benefit than a hazard ratio alone. Sustained, tapered, or response-guided regimens require repeated treatment and covariate histories rather than a single baseline exposure.

### 6.5 Major sources of bias

The most avoidable error is temporal misalignment. If follow-up begins at intensive care unit admission but exposure is defined by corticosteroid receipt during the next 24 h, treated patients must survive long enough to receive therapy, producing immortal time bias. Worsening shock also drives both treatment and mortality, creating confounding by indication; this limits early-versus-late observational comparisons even after multivariable or propensity adjustment (Sacha et al., 2021; Ragoonanan et al., 2022; Zhang et al., 2023; Ahn et al., 2026). Common time zero, explicit grace-period handling, and rich pretreatment covariates reduce these biases but cannot eliminate unmeasured clinician judgment. For trajectory-stratified analyses, positivity should also be examined within each candidate trajectory group, because a subgroup in which nearly all patients receive—or nearly all avoid—corticosteroids cannot support a well-grounded within-group treatment contrast (Hernán et al., 2025; Cole and Hernan, 2008).

Longitudinal strategies introduce exposure-affected time-varying confounding because prior corticosteroids may change vasopressor requirements or lactate, which then influence subsequent treatment and outcome. Marginal structural models or other g-methods may be required, with transparent weight, balance, overlap, and effective-sample-size diagnostics (Cole and Hernan, 2008; Robins et al., 2000). A trajectory-specific form of the same problem arises when class membership is constructed from post-treatment measurements: conditioning on such a treatment-affected phenotype can convert a response variable into an apparently “baseline” subgroup and distort treatment-effect interpretation (Robins et al., 2000). Additional threats include using post-treatment trajectories as baseline subgroups, mixing different molecules and regimens under a generic corticosteroid label, limited positivity, inaccurate medication timestamps, clinically triggered measurement, informative censoring, and death as a competing event. These problems must be addressed primarily through design restriction and explicit treatment and outcome definitions.

### 6.6 Interpretation boundary and translational role

A 2025 multicenter corticosteroid emulation illustrates both the promise and the limits of combining trajectory prediction with causal design (Rajendran et al., 2025). It used early treatment windows and multiple sensitivity analyses, but trajectory-specific estimates varied across cohorts, the treatment definition combined heterogeneous versions, trajectory labels could be influenced by early treatment, and residual confounding remained. The study is therefore proof of concept rather than a validated precision-prescribing rule. Accordingly, the reported trajectory strata should not be interpreted as validated corticosteroid-response biomarkers or as indications to administer or withhold corticosteroids in individual patients (Rajendran et al., 2025). Prediction and causal evaluation answer different questions. A trajectory model estimates a future course; a target trial compares outcomes under alternative implementable strategies. Target trial emulation can reduce avoidable design bias, but it cannot repair unmeasured confounding, measurement error, limited positivity, or ambiguous treatment versions (Hernán et al., 2025). Nor does the label “target trial emulation” confer the evidentiary status of randomization: causal interpretation still depends on the adequacy of the observational data and assumptions including consistency, exchangeability, positivity, and appropriate mapping of the target protocol to those data (Hernán and Robins, 2016; Hernán et al., 2025). These boundaries also constrain the clinical claims that can be drawn from observational emulation. An emulation comparing earlier versus deferred corticosteroid initiation may estimate that prespecified strategy contrast under stated causal assumptions, but it cannot by itself establish that earlier treatment is superior. Likewise, differences in treatment estimates across predicted or observed trajectories do not establish that a trajectory predicts corticosteroid benefit unless treatment-effect modification is prespecified, formally tested, and independently replicated. Finally, analyses that combine heterogeneous corticosteroid exposures cannot establish that hydrocortisone, hydrocortisone plus fludrocortisone, or any other specific regimen should be preferred unless those treatment versions are explicitly distinguished and directly compared. Observational target-trial emulation should therefore be used to sharpen causal questions and generate treatment-strategy hypotheses rather than to convert nonrandomized estimates into definitive prescribing recommendations (Hernán and Robins, 2016; Hernán et al., 2025). Before trajectory-guided corticosteroid use is clinically justified, the subgroup must be defined before exposure, prespecified, reproducible, associated with a replicated treatment interaction, and tested in a prospective enrichment trial. The required temporal specifications and multidimensional outcome domains are summarized in Table 6.

**TABLE 6:** Temporal design and multidimensional outcome framework.

Design or outcome element	Corticosteroid-specific specification	Primary analytical role	Key bias or interpretive risk	Recommended design or analysis	Interpretation boundary
Part A. Temporal and causal design					
Eligibility and time zero	First time an adult with Sepsis-3 septic shock remains vasopressor dependent after initial resuscitation and infection management, while initiation and deferral remain plausible	Align eligibility, strategy assignment, and follow-up	Using ICU admission for controls and drug administration for treated patients	Common decision anchor; explicit timeline diagram; sensitivity analyses using alternative sustained-shock definitions	A proposed research anchor, not a validated universal trigger
Pre-exposure feature window	Time zero must follow all variables used for enrichment or baseline adjustment: prior NEE trajectory, MAP, lactate, SOFA, infection source, support and co-interventions	Eligibility, prognostic/predictive enrichment, baseline confounding control	Post-treatment leakage, care-intensity bias, use of future worst values	Prespecify window, aggregation, missingness, measurement density and timestamp semantics	Only information available before strategy assignment can support pretreatment selection
Treatment strategies and versions	Example: initiate IV hydrocortisone 200 mg/day within 6 h versus no initiation within 6 h; analyze fludrocortisone, other corticosteroids, doses and routes separately	Define an implementable causal contrast	Mixing molecules, dose levels, routes, co-therapy and durations under corticosteroid use	Restrict or explicitly compare treatment versions; report conversions without assuming pharmacological equivalence	Regimen-level effects do not isolate component-level effects
Grace period and delayed initiation	Allow treatment to start during a prespecified 0–6 h window	Emulate realistic clinical delay	Immortal time and future-treatment classification	Clone-censor-weight or sequentially nested trials; censor at deviation and use IPCW with clustered variance	A grace period requires design-based handling; simple end-of-window grouping is biased
Assignment emulation and overlap	Estimate treatment probability from time-zero covariates and assess whether both strategies occur in comparable patients	Approximate baseline exchangeability	Confounding by indication, limited positivity, extreme weights	Standardization/IPTW/TMLE or matching; balance, overlap, weight and effective-sample-size diagnostics	Adjustment cannot address unmeasured determinants or unsupported regions
Sustained and response-guided strategies	Examples: fixed 7-day course, continue until vasopressor liberation, taper after response, or discontinue for toxicity	Estimate longitudinal treatment regimes	Exposure-affected time-varying confounding and informative strategy deviation	Repeated decision points; MSM/IPTW, g-formula or other g-methods; IPCW for deviation	A single baseline exposure model is insufficient for dynamic duration or withdrawal questions
Follow-up and estimands	Follow from common time zero to day 28; assess early shock outcomes through day 7	Estimate interpretable benefit and harm	Different clocks for treated and untreated groups; HR-only interpretation	Risk difference/ratio, RMST, shock-reversal probability and free-days; ITT analogue plus per-protocol analysis	Each estimand answers a distinct clinical question
Reporting and diagnostics	Report target protocol, observational mapping, timeline, causal diagram, timestamps, balance, positivity, weights, censoring and sensitivity analyses	Transparency and reproducibility	Labeling an analysis TTE without a protocol or diagnostics	Use TARGET 2025 for reporting and a separate risk-of-bias assessment; prespecify quantitative sensitivity analysis for residual confounding, such as E-values or other quantitative bias analyses where appropriate, and use negative-control analyses when feasible (Cashin et al., 2025; VanderWeele and Ding, 2017)	Reporting completeness does not establish causal validity. Sensitivity analyses quantify robustness to specified confounding scenarios but cannot demonstrate the absence of unmeasured confounding
Part B. Multidimensional outcome domains					
Hemodynamic response	MAP response, NEE change, time to shock reversal and vasopressor-free days	Pharmacodynamic and early clinical response	Post-treatment response reused as baseline enrichment; death before reversal	Anchor to first administration or common time zero as appropriate; competing-event or composite analysis	More rapid shock reversal does not automatically imply survival benefit
Organ-support outcomes	Ventilation-free days, organ-support-free days and post-treatment SOFA trajectory	Broader physiological and resource-use benefit	Free-days metrics combine survival and support duration; treatment affects measurement opportunity	Define scoring for death and discharge; report component outcomes and sensitivity analyses	Composite measures require transparent decomposition
Kidney outcomes	Incident/progressive AKI, kidney replacement therapy, kidney recovery and MAKE30/90 where appropriate	Organ-specific benefit–harm evaluation	Competing death, indication for KRT, fluid/diuretic co-interventions and ascertainment timing	KDIGO-consistent definitions, competing-risk analysis and separation of perfusion-mediated from direct drug effects	Kidney outcomes remain illustrative, not established corticosteroid efficacy markers
Safety and toxicity	Hyperglycemia, hypernatremia, infection, neuromuscular weakness and other prespecified harms	Net-benefit and treatment-monitoring evaluation	Different ascertainment frequency, baseline susceptibility and competing death	Prespecified definitions, repeated laboratory measures, susceptibility strata and adjudicated clinical events when feasible	Laboratory signals and clinical adverse outcomes are not automatically qualified biomarkers
Mortality and patient-centered outcomes	28- and 90-day mortality, hospital survival and functional recovery when available	Definitive clinical benefit–harm assessment	Residual confounding, loss to follow-up and overinterpretation of subgroup estimates	Absolute effects with uncertainty; interaction analyses prespecified and externally replicated	Observational TTE estimates remain conditional on causal assumptions and require prospective validation

Author-proposed framework retaining detailed temporal and causal specifications. The illustrative 6-h grace period and hydrocortisone 200 mg/day regimen are research specifications, not validated universal treatment thresholds.

Abbreviations: AKI, acute kidney injury; ICU, intensive care unit; IPCW, inverse-probability-of-censoring weighting; IPTW, inverse-probability-of-treatment weighting; KRT, kidney replacement therapy; MAKE, major adverse kidney events; MAP, mean arterial pressure; MSM, marginal structural model; NEE, norepinephrine-equivalent dose; RMST, restricted mean survival time; SOFA, sequential organ failure assessment; TARGET, transparent reporting of observational studies emulating a target trial; TMLE, targeted maximum likelihood estimation.

(1) Time zero must align eligibility, strategy assignment, and follow-up. (2) Post-exposure response variables cannot be reused as pretreatment predictive markers. (3) The example initiation window should be varied in sensitivity analyses. (4) Observational emulation remains dependent on causal and measurement assumptions.

## 7 Organ-specific benefit–harm outcomes

### 7.1 Why mortality alone is insufficient

Mortality remains the most consequential endpoint in septic shock, but it does not fully describe the pharmacological value of corticosteroids. Across major trials, the most reproducible benefit has been faster shock reversal and earlier vasopressor liberation, whereas mortality effects have varied by trial population and regimen (Venkatesh et al., 2018; Annane et al., 2018; Gordon et al., 2016). Evaluation should therefore extend beyond a binary survival endpoint to circulatory support, respiratory support, global organ dysfunction, kidney support, treatment-related toxicity, and longer-term patient-centered outcomes. These domains are complementary rather than interchangeable: an early hemodynamic response should not be treated as a validated surrogate for survival or durable organ recovery.

### 7.2 Hemodynamic, respiratory, and global organ-support outcomes

Hemodynamic endpoints are the most consistently responsive organ-support measures. ADRENAL showed faster shock resolution with hydrocortisone, and APROCCHSS reported more vasopressor-free and organ-failure-free days with hydrocortisone plus fludrocortisone (Venkatesh et al., 2018; Annane et al., 2018). Respiratory findings were less uniform. In ADRENAL, hydrocortisone shortened the initial episode of mechanical ventilation from a median of 7 to 6 days, but did not significantly increase days alive and free of mechanical ventilation when recurrent ventilation was considered (Venkatesh et al., 2018). In APROCCHSS, the difference in ventilator-free days was not statistically significant despite improvements in other organ-support domains (Annane et al., 2018).

Free-day outcomes combine survival with the duration of organ support and should not be interpreted as pure duration outcomes. A higher number of ventilator-, vasopressor-, or organ-support-free days may reflect lower mortality, shorter support among survivors, or both. Death precludes subsequent liberation from organ support and therefore forms part of the competing-event structure rather than a neutral missing observation; recurrent support, transfer or discharge, and the prespecified follow-up horizon can further alter the resulting metric (Yehya et al., 2019). Free-day outcomes should therefore be interpreted alongside mortality, the duration of the initial support episode, recurrent support where relevant, and other component-specific outcomes, with the handling of death and recurrent support explicitly defined. Global measures such as SOFA change or organ-failure-free days may capture multisystem recovery, but they are also treatment-coupled and should not be interpreted as independent evidence of biological recovery or as validated surrogates for survival.

### 7.3 Treatment-related harms

Organ-support benefits must be balanced against metabolic, infectious, and neuromuscular harms. The most consistent safety signal is hyperglycemia; pooled randomized evidence also indicates an increased risk of hypernatremia (Chaudhuri et al., 2024b). These events are clinically relevant because they may increase monitoring and treatment burden and may interact with fluid, electrolyte, and insulin management. By contrast, pooled evidence suggests little or no overall increase in secondary infection, although infection definitions and trial-level estimates remain heterogeneous (Sprung et al., 2008; Chaudhuri et al., 2024b). Neuromuscular weakness is biologically plausible and patient-important, but estimates with contemporary low-dose regimens remain imprecise (Chaudhuri et al., 2024a; Pitre et al., 2024). Safety evaluation should distinguish pretreatment susceptibility, early post-exposure laboratory abnormalities, and later clinical adverse events rather than combine them into an unweighted toxicity composite.

### 7.4 Kidney outcomes as an illustrative case

Sepsis-associated acute kidney injury is common and clinically consequential, making it a useful test case for organ-specific benefit–harm assessment rather than a second independent review topic (Liu J. et al., 2020). SA-AKI was selected as an illustrative organ domain because renal dysfunction in septic shock integrates hemodynamic, microcirculatory, inflammatory, metabolic, and treatment-related processes while providing standardized endpoints spanning KDIGO severity, KRT, recovery, and MAKE. Corticosteroids may influence kidney outcomes indirectly through shock resolution, vasopressor requirements, and inflammatory modulation, but direct human renal protection has not been established.

The parent ADRENAL trial did not demonstrate a significant overall reduction in kidney replacement therapy (KRT), and VANISH found no clear hydrocortisone-related differences in creatinine, urine output, kidney failure, KRT, or mortality (Venkatesh et al., 2018; Gordon et al., 2016). A 2025 *post hoc* analysis of ADRENAL subsequently reported less new KRT with hydrocortisone among patients not receiving KRT at baseline: 21% versus 24%, with an adjusted odds ratio of 0.79 (Donaldson et al., 2025). This is a promising incident-KRT signal, but it does not establish prevention of all SA-AKI. KRT initiation was not protocolized, non-dialysis AKI was not captured, and hydrocortisone did not improve days alive and free of KRT among patients who subsequently commenced KRT (Donaldson et al., 2025).

Future corticosteroid studies may prespecify incident KDIGO AKI, persistent AKI, new KRT, KRT-free days, kidney recovery, and MAKE30/90 (Chawla et al., 2017; KDigo, 2012; Levey et al., 2020; Semler et al., 2016). Definitions should specify baseline kidney function, creatinine and urine-output components, time anchors, death handling, and individual MAKE components. General urine-output trajectory, CRRT lactate, persistent-SA-AKI prediction, and MAKE prediction studies should not be presented as direct corticosteroid treatment-effect evidence.

### 7.5 Integrated interpretation

Corticosteroid benefit should be interpreted across time: early pharmacodynamic response, short-term organ-support liberation, organ injury or recovery, treatment toxicity, and longer-term survival and function. ADRENAL survivor follow-up found no 6-month health-related quality-of-life advantage despite faster shock reversal (Hammond et al., 2020). No organ-specific measure is currently validated as a corticosteroid-specific surrogate endpoint or prescribing biomarker. The appropriate output is therefore a multidimensional benefit–harm profile, with absolute effects and component outcomes reported over prespecified windows, rather than a single responder label.

## 8 From precision evidence to clinician-supervised decision support

Clinical decision support is considered here as a future implementation layer rather than a source of new pharmacological or causal evidence (Lekadir et al., 2025). A corticosteroid-specific CDSS would become clinically relevant only after its underlying prediction or treatment-effect models and the human-system workflow through which they are delivered have undergone external and prospective validation (Lekadir et al., 2025; Vasey et al., 2022).

### 8.1 From validated evidence to action

A future precision corticosteroid CDSS should not be treated as an autonomous prescriber or as a substitute for causal evidence (Lekadir et al., 2025). Once the underlying evidence has been prospectively validated, its output could be linked to a time-stamped, clinician-supervised action: initiate, defer, reassess, monitor for benefit, monitor for harm, or discontinue/taper (Lekadir et al., 2025; Vasey et al., 2022). Any such actions should remain constrained by guideline-based eligibility, a defined decision time, and information available before corticosteroid exposure. A defer recommendation should be explicitly time-limited, whereas reassessment should prompt review of source control, volume status, alternative shock mechanisms, and the original treatment indication rather than automatic treatment escalation.

### 8.2 AI and reinforcement learning: potential and boundaries

The AI Clinician provides an influential methodological example of reinforcement learning (RL) for sequential treatment decisions in sepsis. It learned policies for intravenous fluid and vasopressor dosing from retrospective intensive-care data, but it did not evaluate corticosteroid treatment and therefore should not be interpreted as evidence for corticosteroid-specific decision support (Komorowski et al., 2018).

Corticosteroid-specific machine-learning research has instead estimated individual treatment effects using pooled randomized-trial data, illustrating a supervised treatment-effect modeling approach that is more directly aligned with the question of treatment selection than generic sepsis RL (Pirracchio et al., 2020). However, such retrospective model development and external validation do not by themselves establish prospective clinical utility or a deployable corticosteroid CDSS (Vasey et al., 2022; Pirracchio et al., 2020).

RL is conceptually most relevant when the intervention consists of repeated, state-dependent decisions for which clinically defensible states, actions, and reward functions can be specified (Liu S. et al., 2020; Gottesman et al., 2019). For corticosteroid therapy, such a framework might eventually address genuinely sequential questions such as continuation, tapering, discontinuation for toxicity, or response-guided strategy adjustment rather than simply reproducing a one-time initiation classifier (Liu S. et al., 2020).

At present, reward specification, hidden confounding in retrospective treatment trajectories, incomplete coverage of state–action combinations, off-policy evaluation, and unsafe extrapolation remain major challenges for clinical RL (Gottesman et al., 2019; Festor et al., 2022). Accordingly, near-term corticosteroid CDSS research is better framed around externally validated prediction or treatment-effect models embedded within explicit clinician-supervised decision rules, whereas RL should remain an investigational approach for prespecified sequential treatment strategies (Liu S. et al., 2020; Festor et al., 2022).

### 8.3 Validation beyond discrimination

Internal discrimination is insufficient for clinical deployment. The validation target should match the intended function of the model: discrimination and calibration for outcome prediction, treatment-effect and decision-curve evaluation for individualized-benefit models, and policy-value and safety assessment for RL systems (Pirracchio et al., 2020; Gottesman et al., 2019). Evaluation should include calibration at the intended decision horizon, temporal and external performance, subgroup performance, missingness, computational latency, positive predictive value, alert burden, and clinical utility. Action thresholds should reflect the relative consequences of missed treatment opportunities and unnecessary exposure rather than be selected only by the Youden index. TRIPOD + AI and PROBAST + AI can improve reporting and critical appraisal, but compliance with either framework does not establish transportability or clinical benefit (Collins et al., 2024; Moons et al., 2025). General sepsis-model evaluations illustrate why apparently acceptable discrimination can coexist with poor calibration, low positive predictive value, and substantial alert burden (Wong et al., 2026).

### 8.4 Safety, human oversight, equity, and auditability

Clinical performance should be evaluated at the level of the human-AI team. A clinically useful recommendation needs a timestamp, a clear indication, and an explicit trigger for reassessment. Clinician acceptance or override should be recorded because the performance of the deployed system depends on both the model and how its recommendations are used (Vasey et al., 2022; U. S. Food and Drug Adminstration, 2024; International Medical Device Regulators Forum, 2025). Safety assurance should begin before deployment by defining foreseeable clinical hazards, unsafe action scenarios, and system-level constraints rather than relying only on retrospective performance metrics (Festor et al., 2022). In the AI Clinician safety case, predefined hazardous scenarios were used to constrain the action space, and the reward function was modified to satisfy explicit safety constraints, illustrating that safety can be engineered into a treatment policy rather than assessed only after deployment (Festor et al., 2022). For corticosteroid-specific CDSS, candidate safeguards could include preventing recommendations outside guideline-defined eligibility, blocking post-exposure information from pretreatment selection, flagging missing or out-of-distribution inputs, and requiring clinician review or abstention when evidence support is weak (Lekadir et al., 2025; Festor et al., 2022). Equity and transportability also require explicit evaluation. Models developed in high-resource intensive-care datasets may not retain calibration or clinical utility across populations, institutions, measurement practices, and health systems with different resource constraints (Lekadir et al., 2025; Cross et al., 2024). This concern is especially relevant when candidate precision strategies depend on dense longitudinal electronic-health-record data or rapid transcriptomic testing, because unequal access to the required measurements can itself determine who is eligible to receive model-supported recommendations (Lekadir et al., 2025). Subgroup calibration, missingness, recommendation frequency, override rates, and clinically important failure modes should therefore be audited across demographic groups, institutions, and resource settings rather than inferred from overall model performance (Lekadir et al., 2025; Cross et al., 2024).

Patient notification and informed-consent requirements should likewise be treated as use-case-specific governance questions rather than as a universal requirement for every AI-assisted clinical decision (Rose and Shapiro, 2024). One proposed framework distinguishes among no separate notification, notification alone, and formal informed consent according to factors including model autonomy, departure from standard practice, whether the system is patient facing, and the incremental clinical risk introduced by the model (Rose and Shapiro, 2024). For a corticosteroid CDSS that materially contributes to treatment selection, institutions should therefore prespecify when patients or surrogates are informed of the system’s role, limitations, and clinician oversight, with more explicit disclosure warranted as system autonomy or decision-related risk increases (Lekadir et al., 2025; Rose and Shapiro, 2024).

### 8.5 Prospective evaluation and lifecycle monitoring

After offline temporal and external evaluation, a candidate system should proceed through silent-mode testing, small-scale live evaluation, and comparative clinical assessment before controlled deployment. DECIDE-AI provides guidance for early live evaluation, whereas SPIRIT-AI and CONSORT-AI support protocols and reports of comparative trials involving an AI component (Vasey et al., 2022; Rivera et al., 2020; Liu X. et al., 2020). Deployment should include surveillance for calibration drift, input-data changes, alert frequency, override patterns, subgroup performance, and unintended consequences. Model, threshold, or input changes require version control and reevaluation rather than unmanaged continuous updating (Lekadir et al., 2025; U. S. Food and Drug Adminstration, 2024; International Medical Device Regulators Forum, 2025).

### 8.6 Current readiness

To our knowledge, no corticosteroid-specific CDSS has undergone prospective clinical evaluation demonstrating utility for routine prescribing in septic shock. Existing machine-learning studies have estimated corticosteroid treatment effects or candidate responsiveness, but model-development and retrospective validation studies should not be conflated with prospective CDSS effectiveness (Vasey et al., 2022; Pirracchio et al., 2020). General sepsis-alert studies can inform workflow and transportability, but they do not establish the effectiveness of corticosteroid recommendations. The CDSS should therefore be positioned as the outer implementation layer around guideline eligibility, pretreatment pharmacological enrichment, temporal design, and multidimensional benefit–harm monitoring—not as a replacement for those evidence layers or for clinician responsibility. Figure 3 presents this staged, clinician-supervised implementation architecture.

**FIGURE 3:**
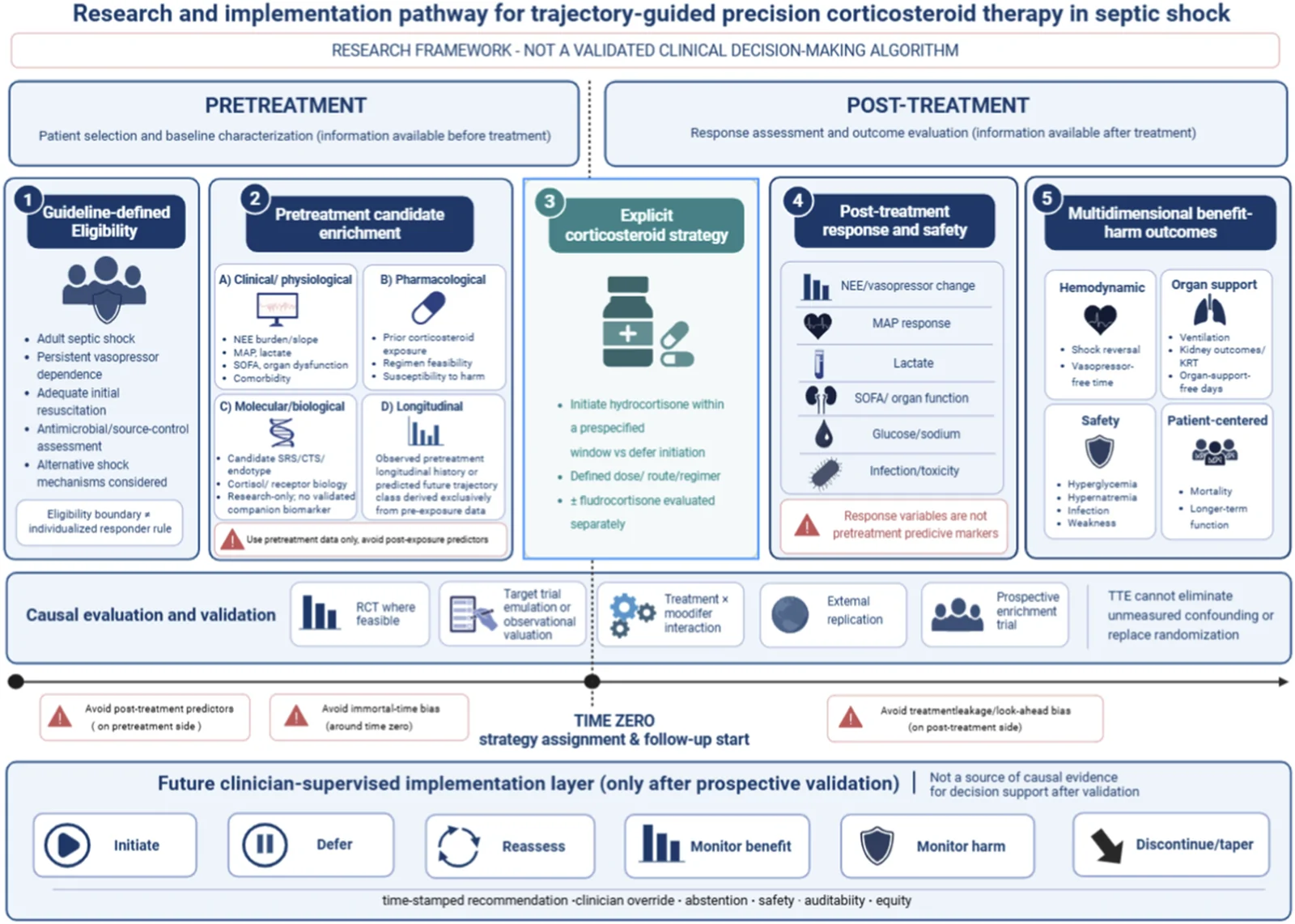
Research and implementation pathway for trajectory-guided precision corticosteroid therapy in septic shock. Guideline eligibility defines entry into the decision pathway. Pretreatment pharmacological, clinical, biomarker, and trajectory information supports candidate enrichment; a defined time zero separates selection from treatment exposure; post-treatment trajectories monitor benefit and harm; and multidimensional outcomes support validation. Clinical decision support forms an outer, clinician-supervised future implementation layer that could translate prospectively validated evidence into initiation, deferral, reassessment, monitoring, or discontinuation/tapering recommendations; it does not itself establish causal treatment benefit. The figure represents a research and validation framework, not a validated clinical decision-making algorithm.

## 9 Discussion

### 9.1 Principal findings and conceptual contribution

The contemporary corticosteroid question in septic shock is no longer adequately framed as whether the drug class is simply effective or ineffective. Across major randomized trials, the most reproducible average treatment effect is accelerated shock reversal and earlier liberation from vasopressors, whereas mortality benefit is not uniform across populations and regimens (Sprung et al., 2008; Venkatesh et al., 2018; Annane et al., 2018). This pattern is pharmacologically coherent: corticosteroids can improve vascular responsiveness and modulate inflammatory signaling, but these same actions may produce metabolic, immune, and neuromuscular harms. Clinically, the decision is time dependent: the expected hemodynamic benefit has to be weighed against treatment-related harm at the point corticosteroids are considered.

The apparent inconsistency of the corticosteroid literature partly reflects the fact that different studies answer different questions. Randomized trials establish regimen-level effects, and their most consistent finding is faster shock reversal. Biomarker and transcriptomic studies instead ask whether treatment effects vary across patients, while trajectory studies describe an evolving clinical state that is itself influenced by treatment. Observational causal analyses address yet another question—when and under what treatment strategy corticosteroids might be beneficial. Keeping these questions separate is essential because prognosis, treatment response, and treatment-effect modification are not interchangeable.

### 9.2 Evidence readiness and current clinical implications

#### 9.2.1 Established clinical evidence

Several conclusions are sufficiently mature to inform current practice. Corticosteroids should be considered adjunctive therapy within the guideline-defined boundary of adult septic shock with persistent hemodynamic instability rather than broad anti-inflammatory therapy for unselected sepsis (Chaudhuri et al., 2024a; Prescott et al., 2026). Across contemporary randomized trials, the most consistent near-term effect is faster shock reversal and earlier liberation from vasopressors, whereas mortality effects vary according to the tested population and treatment regimen (Sprung et al., 2008; Venkatesh et al., 2018; Annane et al., 2018). High-dose, short-course anti-inflammatory regimens are not supported, and prophylactic hydrocortisone before septic shock develops has not demonstrated clinical benefit (Chaudhuri et al., 2024a; Keh et al., 2016; Sprung et al., 1984; Bone et al., 1987). Hyperglycemia and hypernatremia remain among the most consistently supported treatment-related harms (Chaudhuri et al., 2024a; Sprung et al., 2008; Annane et al., 2018). These findings define population-level eligibility and the effects of specific tested regimens, but they do not constitute an individualized treatment-selection rule.

#### 9.2.2 Supported but uncertain evidence

Several clinically important questions have biological, pharmacological, or trial-level support but remain insufficiently resolved for individualized prescribing. Alterations in cortisol availability, glucocorticoid-receptor biology, prereceptor metabolism, and tissue responsiveness provide plausible explanations for interindividual variation, yet no cortisol, corticotropin, receptor, or related mechanistic threshold has been validated to identify individual corticosteroid responders (Guerrero et al., 2013; Vardas et al., 2017; Teblick et al., 2022; Annane et al., 2017; Hamrahian et al., 2004; Boonen et al., 2013; Venkatesh et al., 2007). The optimal initiation threshold, administration mode, dose–exposure relationship, treatment duration, and withdrawal strategy likewise remain uncertain (Chaudhuri et al., 2024a; Prescott et al., 2026; Sprung et al., 2008; Venkatesh et al., 2018; Annane et al., 2018; Pitre et al., 2024; Loisa et al., 2007; Gordon et al., 2016). The incremental contribution of fludrocortisone is also unresolved: APROCCHSS established efficacy of the complete regimen of hydrocortisone plus fludrocortisone against placebo, whereas COIITSS directly but inconclusively evaluated fludrocortisone on a common hydrocortisone background, and cross-trial comparison with ADRENAL cannot isolate a component effect (Venkatesh et al., 2018; Annane et al., 2018; COIITSS Study Investigators, 2010). These questions are therefore clinically relevant and biologically plausible, but they should not be converted into individualized treatment rules without further direct comparative validation.

#### 9.2.3 Hypothesis-generating evidence

Clinical severity, transcriptomic endotypes, longitudinal trajectories, and observational causal analyses may identify candidate subgroups in whom corticosteroid benefit or harm differs, but current evidence remains insufficient for routine treatment selection. Higher baseline risk may increase absolute benefit without demonstrating modification of the relative treatment effect, and prognostic enrichment should therefore not be interpreted as predictive enrichment in the absence of a formal treatment-by-marker interaction (Iwashyna et al., 2015; Stanski and Wong, 2020). Transcriptomic classifications including SRS1/SRS2, pediatric Endotype A/B, IA-P/IN-P, and consensus transcriptomic subtypes were developed in different populations and platforms and should not be regarded as interchangeable (Antcliffe et al., 2019; Wong et al., 2016; Venkatesh et al., 2025; Sciclun et al., 2025). The corrected VANISH analysis preserved a hydrocortisone-by-SRS interaction signal, but substantial prior misclassification, subgroup uncertainty, and the absence of a prospective SRS-guided trial preclude using these findings as an individual treatment rule (Antcliffe et al., 2019; Antcliffe et al., 2022). Pediatric Endotype A/B findings and their exploratory transport to adults remain similarly hypothesis-generating (Wong et al., 2015; Wong et al., 2017; Wong et al., 2018; Wong et al., 2021), while the ADRENAL transcriptomic substudy did not demonstrate clear heterogeneity in its primary comparison (Sciclun et al., 2025; U. S. Food and Drug Adminstration, 2021). Consensus transcriptomic subtypes improve cross-study biological harmonization, but the observed CTS2 corticosteroid-associated harm signal did not establish a statistically significant hydrocortisone-by-CTS interaction in the VANISH reanalysis (Sciclun et al., 2025). Longitudinal organ-dysfunction, lactate, and hemodynamic trajectories can improve phenotypic characterization (Xu et al., 2022; Ma et al., 2026; Shen et al., 2026; Leng et al., 2024), but treatment-affected or post-exposure trajectories cannot be retrospectively reclassified as pretreatment predictors of corticosteroid benefit (Hernán and Robins, 2016; Hernán et al., 2025; Robins et al., 2000). Observational target-trial emulation can improve alignment of eligibility, treatment assignment, time zero, and follow-up, but it cannot eliminate unmeasured confounding, measurement error, limited positivity, or ambiguity in treatment versions (Hernán and Robins, 2016; Hernán et al., 2025; Robins and Finkelstein, 2000; Cain et al., 2010; Cole and Hernan, 2008; Robins et al., 2000). These approaches should therefore be regarded as sources of testable treatment-effect hypotheses rather than established prescribing evidence.

#### 9.2.4 Proposed research and implementation framework

Trajectory-guided enrichment, prospectively validated treatment-effect prediction, multimodal modeling, target-trial design principles, and clinician-supervised decision support represent a research pathway rather than an emerging bedside algorithm. Their role is to specify how candidate treatment-effect modifiers can be defined before exposure, tested prospectively, evaluated across multidimensional benefit–harm outcomes, and ultimately translated into clinically interpretable actions if sufficient validation is achieved (Hernán and Robins, 2016; Hernán et al., 2025; Cashin et al., 2025). Clinical decision support and AI should therefore remain implementation layers around validated evidence rather than independent sources of pharmacological or causal evidence. No corticosteroid-specific CDSS has yet demonstrated prospective clinical utility sufficient for routine prescribing in septic shock, and retrospective treatment-effect or machine-learning models should not be conflated with prospectively validated decision-support systems (Vasey et al., 2022; Pirracchio et al., 2020). Accordingly, increasing analytical complexity should not be interpreted as increasing evidentiary strength; trajectory-guided selection, target-trial-based observational analyses, and AI-supported implementation remain frameworks for validation and future clinical translation rather than evidence that individualized corticosteroid treatment improves outcomes.

### 9.3 Translational implications

Candidate precision strategies must preserve the distinction between prognosis, prediction, pharmacodynamic response, and safety. Transcriptomic signals such as SRS1/SRS2, pediatric Endotype A/B, and IA-P/IN-P provide biologically coherent hypotheses, but none is ready for routine treatment selection. Their classifiers are not interchangeable, several findings arise from secondary or exploratory analyses, and the overall ADRENAL transcriptomic comparison did not demonstrate clear heterogeneity (Antcliffe et al., 2019; Venkatesh et al., 2025; Antcliffe et al., 2022; Wong et al., 2015; Wong et al., 2017; Wong et al., 2018; Wong et al., 2021; Cohen et al., 2021). Future predictive enrichment requires a pretreatment assay that is analytically valid, rapidly available within the clinical decision window, prospectively specified, and independently replicated with a formal treatment-by-marker interaction.

Dynamic clinical trajectories may complement molecular enrichment by describing whether shock burden and organ dysfunction are improving, persisting, or worsening. However, these trajectories frequently reflect both disease evolution and treatment response. These trajectories partly reflect prior treatment and are best viewed as treatment-coupled clinical phenotypes; they should not be assumed to represent stable underlying biology. Variables used for treatment selection must end before a defined time zero, while post-exposure changes in vasopressor dose, mean arterial pressure, lactate, and organ support belong to response or safety monitoring. Target trial emulation can improve alignment of eligibility, strategy assignment, and follow-up and can reduce avoidable temporal bias, but it cannot eliminate unmeasured confounding, limited positivity, treatment-version ambiguity, or measurement error (Hernán and Robins, 2016; Hernán et al., 2025; Robins and Finkelstein, 2000; Cain et al., 2010; Cole and Hernan, 2008; Robins et al., 2000; Hernán et al., 2016).

The same discipline applies to outcomes and implementation. Precision evaluation requires a multidimensional profile that includes shock reversal, organ-support liberation, metabolic and infectious harms, mortality, and longer-term function. Kidney outcomes illustrate the value of standardized organ-specific validation, but current evidence does not establish corticosteroid-specific renal protection. A clinical decision support system should therefore sit outside these evidence layers as a clinician-supervised implementation mechanism. It should translate validated evidence into actions such as initiate, defer, reassess, monitor, or discontinue, rather than generate new pharmacological evidence by itself.

### 9.4 Research priorities

A central unresolved question is whether any pretreatment feature can reproducibly identify patients with differential benefit or harm from corticosteroids. Rapid transcriptomic or biomarker assays therefore require prospective evaluation in enriched or stratified randomized trials, with the same assay, threshold, treatment strategy, and interaction hypothesis carried from development into validation. Both absolute and relative treatment effects are important, because the clinical value of an enrichment strategy depends not only on relative effect modification but also on baseline risk and the magnitude of potential benefit and harm.

Treatment timing and regimen design require equally explicit evaluation. Trials and embedded platform studies need prespecified eligibility criteria, a common time zero, pretreatment feature windows, initiation strategies, permitted grace periods, and follow-up before analysis. Observational target-trial emulations may help refine hypotheses about early versus delayed initiation, but they cannot replace prospective randomization. Questions of treatment duration also remain open: fixed courses should be compared directly with response-guided continuation, discontinuation, or tapering using designs that accommodate repeated treatment decisions. A dedicated, adequately powered trial comparing hydrocortisone alone with hydrocortisone plus fludrocortisone would therefore be informative. In COIITSS, the fludrocortisone comparison was a secondary objective and was not included in the sample-size calculation (COIITSS Study Investigators, 2010). Future trials would also benefit from harmonized definitions of organ-support and safety outcomes, including death handling in free-day endpoints, new kidney replacement therapy, MAKE components, and kidney recovery.

Clinical implementation becomes relevant only after a treatment rule has shown reproducible clinical validity. Candidate corticosteroid CDSSs can then progress from analytical and external validation to silent-mode testing, early live evaluation, and comparative clinical assessment. At that stage, evaluation should focus on the combined model-workflow system—including clinician uptake, overrides, alert burden, overtreatment, and post-deployment drift—rather than discrimination alone.

### 9.5 Strengths and limitations

This review has several strengths. It integrates pharmacology, randomized trial evidence, treatment-effect heterogeneity, longitudinal clinical data, causal design, organ-specific outcomes, and implementation science within one treatment-focused framework. It also separates clinically supported mechanisms from translational and hypothesis-generating evidence, distinguishes prognostic from predictive enrichment, and makes temporal availability a prerequisite for any candidate precision rule.

The review also has important limitations. It is a structured state-of-the-art narrative review rather than a systematic review or meta-analysis and was not designed to identify every eligible publication or provide pooled estimates. The included evidence is heterogeneous: randomized trials, secondary transcriptomic analyses, observational trajectory studies, causal-methodology papers, and general sepsis-CDSS studies cannot be assigned equal clinical weight. Most candidate effect modifiers remain exploratory, and real-world causal analyses remain vulnerable to unmeasured confounding, treatment-affected labels, timestamp error, and limited overlap. Definitions of organ-support-free days, kidney replacement therapy, MAKE, and recovery also vary across studies. Finally, implementation evidence comes largely from general sepsis-alert systems and informs transportability and workflow requirements rather than corticosteroid effectiveness. These limitations reinforce the central conclusion: trajectory-guided precision corticosteroid therapy is a research pathway that now requires prespecified, prospective, and clinically integrated validation.

## 10 Conclusion

Contemporary low-dose corticosteroid regimens have an established adjunctive role in vasopressor-dependent septic shock, with accelerated shock reversal as their most consistent clinical effect. Mortality effects remain regimen- and population-dependent, and the incremental contribution of fludrocortisone to hydrocortisone remains unresolved. No cortisol-based test, molecular endotype, clinical phenotype, or trajectory-defined subgroup is currently validated to identify individual corticosteroid responders or to mandate treatment selection. Future studies should therefore prioritize prespecified and replicated treatment-effect modifiers, explicit treatment timing and regimen definitions, direct regimen comparisons where feasible, and multidimensional, clinically meaningful benefit–harm outcomes. Target trial emulation can strengthen the design of observational treatment-effect analyses and reduce avoidable temporal bias, but it cannot reproduce randomization or eliminate unmeasured confounding; prospective validation remains necessary before trajectory-guided treatment rules or corticosteroid-specific decision support can inform routine care. Precision corticosteroid therapy should therefore be viewed as a testable research framework for identifying differential benefit–harm profiles, not as a currently validated prescribing algorithm.

## Glossary

ACTH: adrenocorticotropic hormone
ADRENAL: Adjunctive Glucocorticoid Therapy in Patients with Septic Shock trial
AKI: acute kidney injury
APROCCHSS: Activated Protein C and Corticosteroids for Human Septic Shock trial
BEST: Biomarkers, EndpointS, and other Tools
CDSS: clinical decision support system
CI: confidence interval
CIRCI: critical illness-related corticosteroid insufficiency
CORTICUS: Corticosteroid Therapy of Septic Shock trial
GR: glucocorticoid receptor
HYPRESS: Hydrocortisone for Prevention of Septic Shock trial
IA-P: immune adaptive-prevalent
ICU: intensive care unit
IN-P: immune innate-prevalent
IPCW: inverse-probability-of-censoring weighting
IPTW: inverse-probability-of-treatment weighting
KRT: kidney replacement therapy
MAKE: major adverse kidney events
MAP: mean arterial pressure
MR: mineralocorticoid receptor
MSM: marginal structural model
NEE: norepinephrine-equivalent dose
NF-κB: nuclear factor kappa B
RCT: randomized controlled trial
RMST: restricted mean survival time
SA-AKI: sepsis-associated acute kidney injury
SOFA: Sequential Organ Failure Assessment
SRS: sepsis response signature
TARGET: Transparent Reporting of Observational Studies Emulating a Target Trial
TMLE: targeted maximum likelihood estimation
TTE: target trial emulation
VANISH: Vasopressin versus Norepinephrine as Initial Therapy in Septic Shock trial
11β-HSD1: 11β-hydroxysteroid dehydrogenase type 1
AI: artificial intelligence
AP-1: activator protein 1
ARDS: acute respiratory distress syndrome
COIITSS: Corticosteroid Treatment and Intensive Insulin Therapy for Septic Shock trial
CrI: credible interval
CTS: consensus transcriptomic subtype
ESICM: European Society of Intensive Care Medicine
FDA: Food and Drug Administration
FluDReSS: Fludrocortisone Dose-Response in Septic Shock trial
HR: hazard ratio
NIH: National Institutes of Health
OR: odds ratio
PD: pharmacodynamic
PK: pharmacokinetic
ROR: ratio of odds ratios
RR: relative risk
RL: reinforcement learning
SCCM: Society of Critical Care Medicine
